# Can acyclic conformational control be achieved *via* a sulfur–fluorine *gauche* effect?†
†Electronic supplementary information (ESI) available. CCDC 1048074–1048078. For ESI and crystallographic data in CIF or other electronic format see DOI: 10.1039/c5sc00871a


**DOI:** 10.1039/c5sc00871a

**Published:** 2015-04-17

**Authors:** C. Thiehoff, M. C. Holland, C. Daniliuc, K. N. Houk, R. Gilmour

**Affiliations:** a Organisch Chemisches Institut, and Excellence Cluster EXC 1003 , Cells in Motion , Westfälische Wilhelms-Universität Münster , Corrensstrasse 40 , Münster , Germany . Email: ryan.gilmour@uni-muenster.de; b Department of Chemistry and Biochemistry , University of California Los Angeles , 607 Charles E. Young Drive East , Los Angeles 90095-1569 , USA . Email: houk@chem.ucla.edu; c Excellence Cluster EXC 1003 , Cells in Motion , Westfälische Wilhelms-Universität Münster , Münster , Germany

## Abstract

The *gauche* conformation of the 1,2-difluoroethane motif is known to involve stabilising hyperconjugative interactions between donor (bonding, *σ*_C–H_) and acceptor (antibonding, σ*C–F) orbitals. This model rationalises the generic conformational preference of F–C_β_–C_α_–X systems (*φ*_FCCX_ ≈ 60°), where X is an electron deficient substituent containing a Period 2 atom. Little is known about the corresponding Period 3 systems, such as sulfur and phosphorus, where multiple oxidation states are possible. Conformational analyses of β-fluorosulfides, -sulfoxides and -sulfones are disclosed here, thus extending the scope of the fluorine *gauche* effect to the 3rd Period (F–C–C–S(O)_*n*_; *φ*_FCCS_ ≈ 60°). Synergy between experiment and computation has revealed that the *gauche* effect is only pronounced in structures bearing an electropositive vicinal sulfur atom (S^+^–O^–^, SO_2_).

## Abstract 2

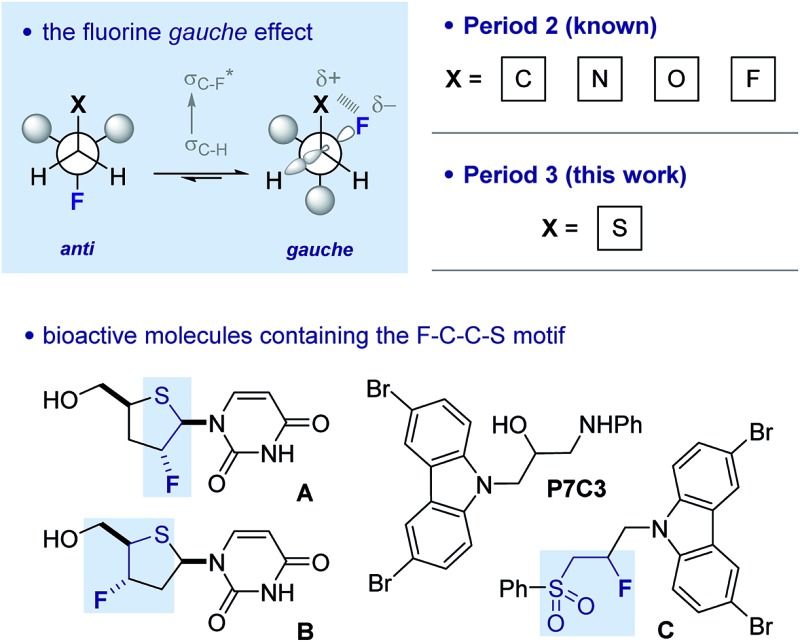
Herein detailed conformational analyses of β-fluorosulfides, -sulfoxides and -sulfones are disclosed, thus extending the scope of the fluorine *gauche* effect to the 3^rd^ Period (X = SR, SOR, SO_2_R; *φ*_FCCS_ ≈ 60°).

## 

Controlling rotation about C(sp^3^)–C(sp^3^) bonds is strategically important in molecular design, not least to determine the spatial positioning of substituents on the component atoms.^1^ Of the various acyclic conformational control strategies in common practice, the fluorine *gauche* effect^2^ has gained momentum in recent years on account of the minimal steric footprint imposed by this substituent; this often leads to conformer populations that are inaccessible by traditional steric locking approaches. The counterintuitive preference of the parent 1,2-difluoroethane scaffold to populate the *gauche* conformer preferentially can be rationalised by invoking hyperconjugative *σ*_C–H_ → σ*C–F interactions. This conformational preference is conserved in a number of F–C–C–X systems where X is electron deficient (Fig. 1).^3^ A simplified donor–acceptor model is didactically valuable in rationalising and predicting conformation, while more detailed analysis reveals that both orbital and electrostatic effects are involved.^4^ This is particularly true when X carries a (partial) positive charge, and electrostatic interactions contribute significantly. The strategic installation of the F–C–C–X motif can lead to predictable molecular topologies on account of the *gauche* effect (*φ*_FCCX_ ≈ 60°): the *caveat* that stereoelectronic effects can be overridden by prevailing steric factors must always be considered.

**Fig. 1:**
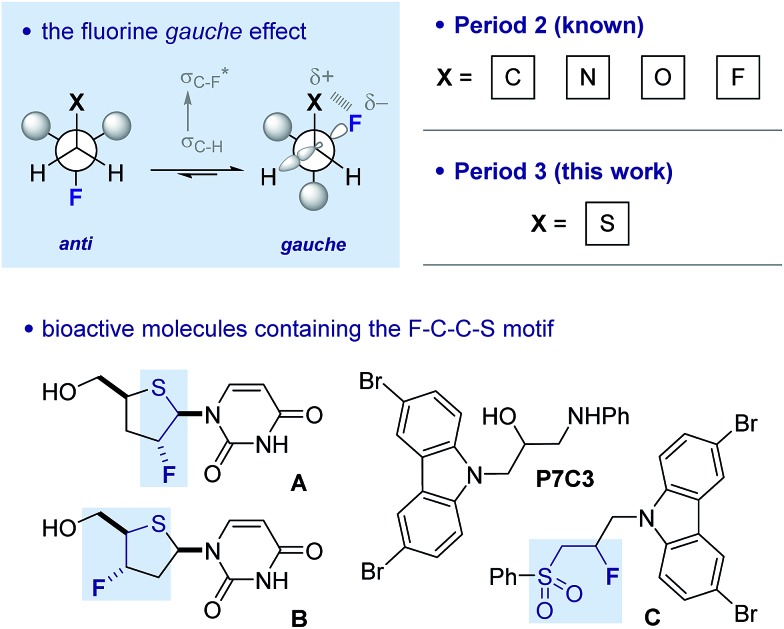
The fluorine *gauche* effect. Selected literature precedence for a potential sulfur–fluorine *gauche* effect. Lower left: fluorinated deoxy-4′-thio pyrimidine nucleosides **A** and **B**.^11^ Lower right: proneurogenic compound P7C3 and its β-fluorinated sulfone derivative (**C**).^12^

This approach to molecular design has found widespread application in catalysis,^5^ bioactive molecule design,^6^ material science^7^ and agrochemistry.^8^ In the majority of cases, the substituent (X) is a Period 2 atom, typically oxygen or nitrogen. In contrast, the manifestation of this phenomenon in combination with 3rd row elements has been largely ignored despite the importance of sulfur and phosphorus containing compounds in industry and academia. Recent interest in the preparation^9^ and properties of compounds containing the F–C–C–S(O)_*n*_ (*n* = 0, 1 and 2) unit prompted this study.

There is limited structural evidence consistent with the postulated sulfur–fluorine *gauche* effect. In the mid-1980s, Carretero and co-workers reported a NMR study of β-fluorinated thioethane derivatives, including various sulfides, sulfoxides, sulfones and sulfonium salts.^10^ Vicinal coupling constant analysis is consistent with a *gauche* orientation of the sulfur and fluorine atoms. Further evidence of this phenomenon derives from the X-ray structure analyses of fluorinated deoxy-4′-thiopyrimidine nucleosides such as **A** and **B** (Fig. 1), where torsional angles of *φ*_FCCS_ ≈ 80° approach the expected stereoelectronic requirements despite the constraints imposed by the ring.^11^ Finally, a recent study by Ready and co-workers identified carbazole P7C3 as displaying potent neuroprotective activity.^12^ A lead structure in this investigation is derivative **C**, containing the β-fluorosulfone unit. Herein we report a combined experimental and computational study of the fluorine–sulfur *gauche* effect with specific emphasis on sulfides, sulfoxides and sulfones.

Our recent interest in the fluorine *gauche* effect in pyrrolidine organocatalysts (X = N)5a,b,f led us to explore tetrahydrothiophene derivatives **2**, **3** and **4** (Scheme 1) as scaffolds for this study. It was envisaged that the diffuse nature of sulfur orbitals, and the polarised nature of the oxidised forms (*e.g.*, S^+^–O^–^, SO_2_) would generate hyperconjugative and/or charge–dipole interactions that might manifest themselves in diagnostic conformations. The heterocycles can exist as *synclinal-endo* and *synclinal-exo* conformers that can easily be distinguished by vicinal (^3^*J*) coupling constant analysis. Structures **2**, **3** and **4** were prepared from the intermediate **1** (Scheme 1).^13^ Direct deoxyfluorination of **1** was facile and furnished **2** in 54% yield. This is noteworthy given the dearth of information of fluorination of this substrate class. Subsequent oxidation to the corresponding sulfoxide **3** proceeded smoothly in 68% yield and with excellent levels of diastereoselectivity (97 : 3), giving a first insight into the possible role of fluorine in influencing the conformation of such systems. Finally, upon exposure to excess *m*CPBA, the sulfone **4** was generated in 85% yield.

**Scheme 1:**
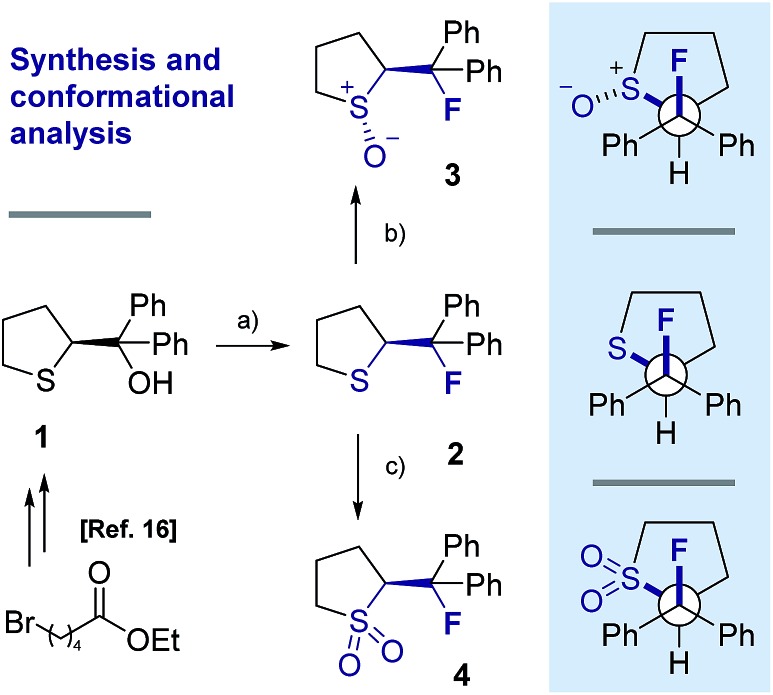
Synthetic route to the cyclic sulfide **2**, sulfoxide **3** and sulfone **4** from **1**. (a) DAST, Na_2_CO_3_, CH_2_Cl_2_, 0 °C to rt, 16 h; (b) *m*CPBA (1.0 eq.), CH_2_Cl_2_, 0 °C to rt, 50 h; (c) *m*CPBA (3.0 eq.), CH_2_Cl_2_, 0 °C to rt, 17 h.

## X-ray crystal structure analysis

**Fig. 2:**
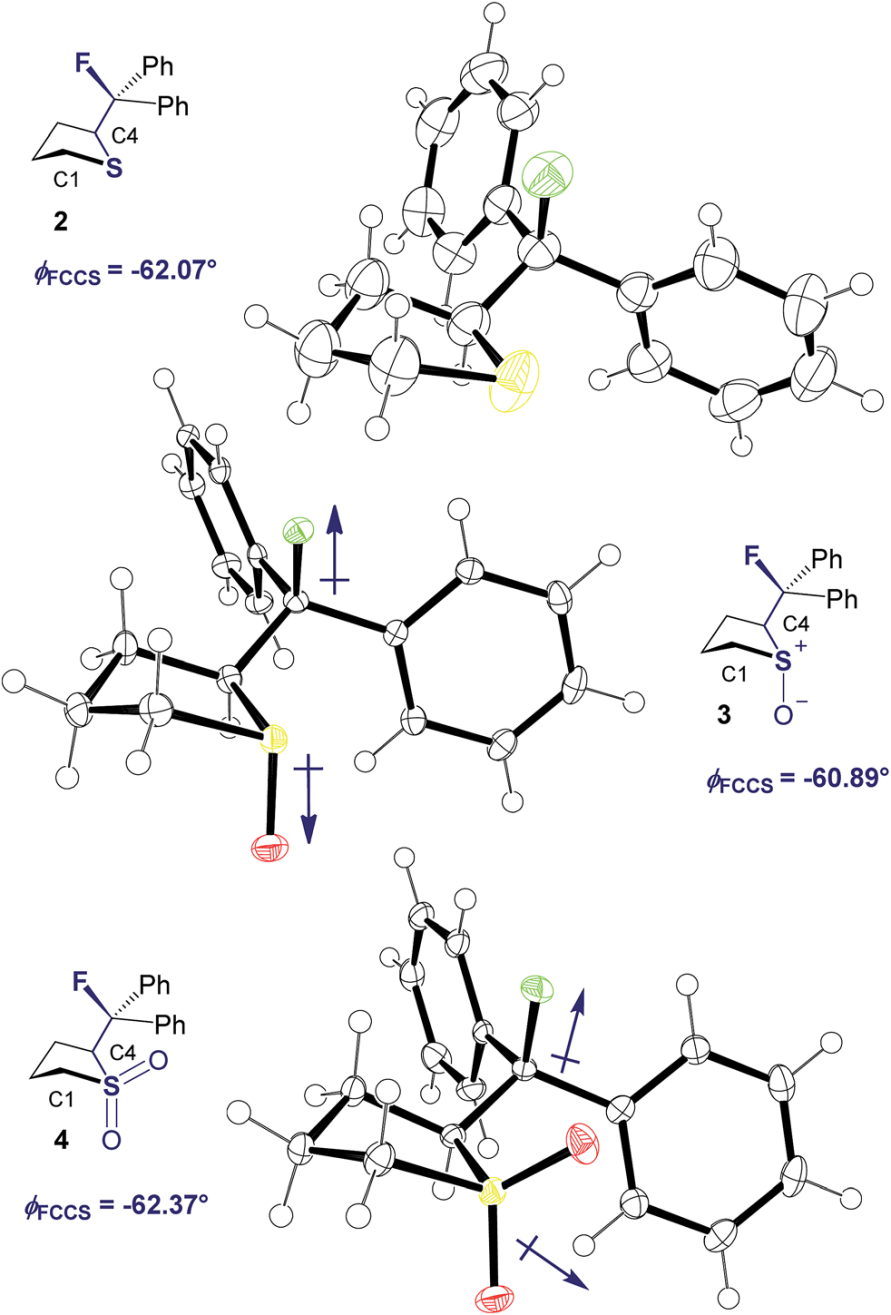
X-ray structural analyses of cyclic compounds **2**, **3** and **4**. Thermal ellipsoids shown at 50% probability level.^14^

Single crystals suitable for X-ray analysis were obtained in all cases (Fig. 2).^14^ Each structure exhibited a *gauche* preference, favouring the *synclincal-endo* conformation (**2**, **3**, **4**, *φ*_FCCS_ = –62.07°, –60.89°, –62.37°, respectively; Table 1). Common to all structures is an unusually long S1–C4 bond length as compared to the S1–C1 bond length (Table 1, Δ*d* ≈ 0.027 Å, 0.039 Å, 0.053 Å, for **2**, **3** and **4**, respectively). To place this observation in context with comparable sulfur containing structures, a selection of C–S bond lengths are provided in Table 1 (right column).^15^ This may be noteworthy in view of the importance of fractional bonds in translating small changes in ground state structures to reactivity.^16^ Importantly, the vicinal C–H and C–F bonds are *antiperiplanar* (179°, –177°, 178.15°) thus allowing for stabilising hyperconjugative interactions (*σ*_C–H_ → σ*C–F), with C–F bond lengths of 1.41 Å, 1.42 Å and 1.42 Å, for **2**, **3** and **4** respectively. The solid state structure of sulfoxide **3**, prepared by diastereoselective oxidation, reveals a conformation in which the C–F and S–O dipoles are minimised. The sulfone derivative **4** preferentially adopts the conformation placing the fluorine atom *synclinal-endo* to the sulfur centre: this minimises repulsion with the non-bonding electron pairs of the oxygen atoms.

**Table 1:** Selected crystallographic data for compounds **2**, **3**, **4**, **6** and **7** (ref. 14)

Compound	*φ*_FCCS_ [°]	*d*_S1–C4_ [Å]	*d*_S1–C1_ [Å]	Δ*d* [Å]	*Literature d*_S–C_ [Å]^15^
**2**	–62.07	1.8379(16)	1.8107(19)	0.027	1.827^*a*^
**3**	–60.89	1.853(2)	1.814(2)	0.039	1.818^*b*^
**4**	–62.37	1.832(2)	1.799(2)	0.054	1.786^*c*^
**6**	–55.87	1.764(9)^*d*^	1.799(6)^*e*^	0.035	1.790^*f*^/1.818^*b*^
**7**	–68.12	1.7789(15)^*d*^	1.7791(15)^*e*^	0.0002	1.763^*g*^/1.786^*c*^

^*a*^In tetrahydrothiophene.

^*b*^In a C–S(O)–C motif.

^*c*^In a C–S(O_2_)–C motif.

^*d*^S–C(sp^3^) bond.

^*e*^S–C(Ar) bond.

^*f*^In C_Ar_–S(O)–C motif.

^*g*^In C_Ar_–S(O_2_)–C motif.

**Scheme 2:**
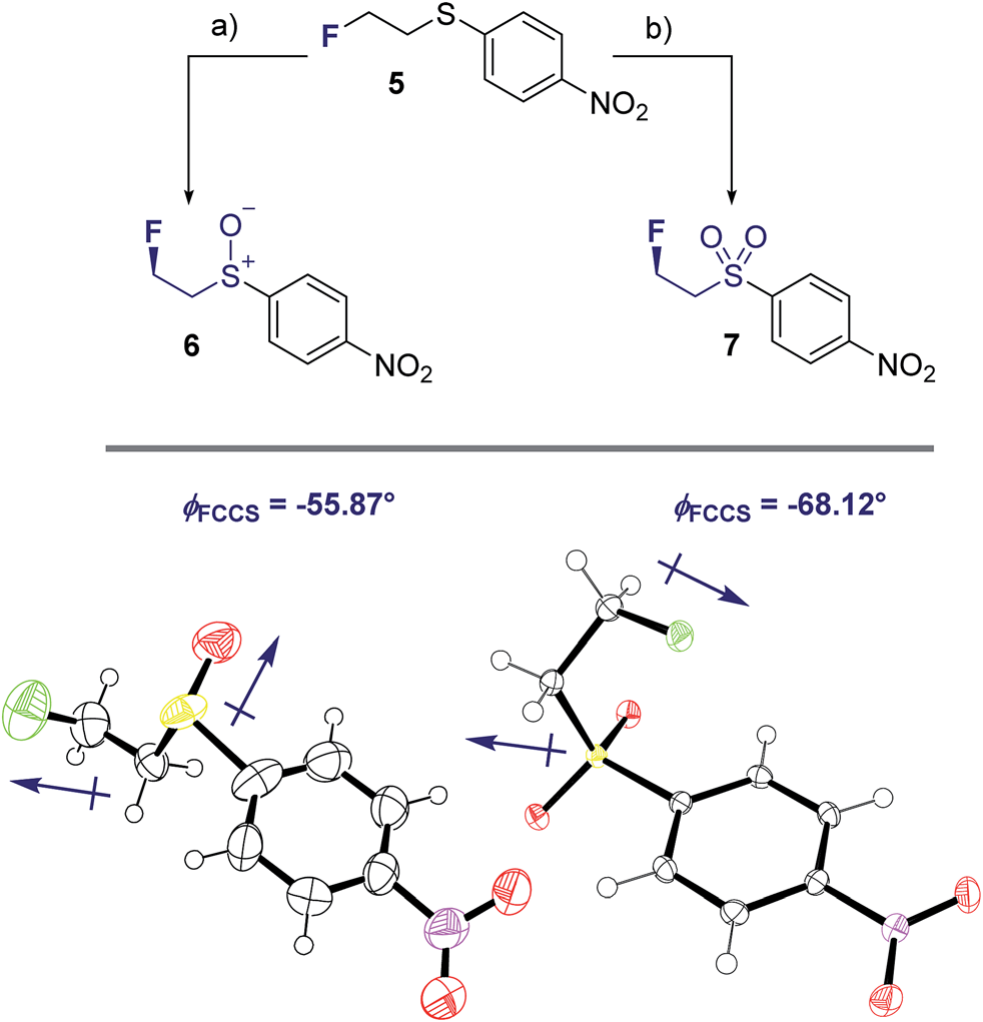
Syntheses and X-ray crystal structure analyses^14^ of linear sulfoxide **6** and sulfone **7**. (a) *m*CPBA (1.0 eq.), CH_2_Cl_2_, 0 °C to rt, 26 h; (b) *m*CPBA (3.0 eq.), CH_2_Cl_2_, 0 °C to rt, 17 h. Thermal ellipsoids shown at 50% probability level.

To ensure that the *gauche* orientation observed in the tetrahydrothiophene derivatives **2**, **3** and **4** is not a consequence of unfavourable non-bonding interactions with the ring, a sterically less demanding, linear system was synthesised for comparison. Reaction of 4-nitrothiophenol with tosylated 2-fluoroethanol^17^ afforded the linear sulfide **5**; this was subsequently converted to sulfoxide **6** and sulfone **7** (Scheme 2). It was possible to grow crystals of compounds **6** and **7** that were suitable for X-ray analysis.^14^ In both cases, the C–S and C–F bonds were oriented in the expected *gauche* arrangement (**6** and **7**, *φ*_FCCS_ = –55.87°, –68.12°, respectively; Table 1). These conformations allow for *σ*_C–H_ → σ*C–F interactions, again indicating that this effect likely is due in part to hyperconjugative stabilisation. Consistent with structure **3** (Fig. 2, centre), the X-ray analysis of sulfoxide **6** reveals a conformation where the C–F and S–O dipoles oppose each other. In sulfone **7**, the C–F bond adopts a *gauche* arrangement that circumvents interaction with the SO_2_ unit.

## NMR solution phase conformational analysis

To complement the solid state investigation, a solution phase NMR conformer population analysis of the cyclic compounds was performed.^18^ Assuming that only staggered conformers with torsion angles of –60° (–*gauche*), 60° (+*gauche*) and 180° (*anti*) contribute significantly to the population in solution phase, the measured coupling constant *J* can be described by the equation *J* = *x*_–g_*J*_–g_ + *x*_+g_*J*_+g_ + *x*_a_*J*_a_. Furthermore, the approximation that the dependency of *J* is symmetrical about 0° (ref. 19) renders the following simplification valid: *J*_–g_ = *J*_+g_ = *J*_g_. Hence, the molar fraction of the *anti* conformer can be determined according to the following expression: *x*_a_ = (*J* – *J*_g_)/(*J*_a_ – *J*_g_).^20^ Whilst literature values for ^3^*J*_CF_ are available (*J*_g_ = 1.2 Hz, *J*_a_ = 11.2 Hz)^21^ the related ^3^*J*_HF_ can be calculated based on a modified Karplus equation.^22^ Inserting the measured ^3^*J* coupling constants (Table 2) allows for the determination of combined populations of the –*gauche* and +*gauche* conformers of >80% in all cases. Comparison of the ^3^*J*_HF_ coupling constants of both series reveals the following trend: upon oxidation of the sulfide to the corresponding sulfoxide, a significant increase in the magnitude of the coupling constant is observed (^3^*J*_HF_ = 29.2, 37.8 and 19.2, 30.0 Hz for **2**, **3** and **5**, **6**, respectively). Further oxidation to the sulfone results in coupling constant values that are only slightly augmented relative to those of the parent sulfides (^3^*J*_HF_ = 31.2 and 23.8 Hz for **4** and **7**, respectively). These analyses reveal that the major solution phase conformers closely resemble the solid state structures determined by crystallography, and are fully consistent with the notion of a sulfur–fluorine *gauche* effect.

**Table 2:** Conformational analysis of cyclic sulfur systems **2**, **3** and **4** in solution phase based on ^3^*J* coupling constant analysis

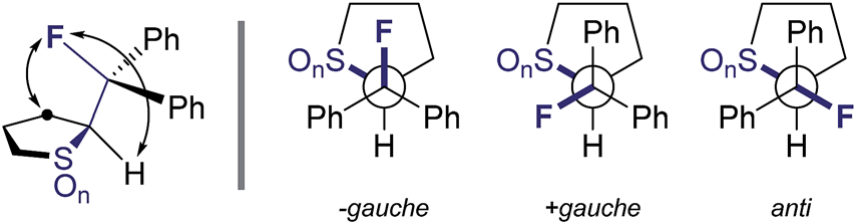					
Compound	^3^ *J*_HF_ [Hz]	^3^ *J*_CF_ [Hz]	–*gauche* [%]	+*gauche* [%]	*anti* [%]
**2** ^*a*^ (*n* = 0)	29.2	3.2	61.3	20.0	18.7
**3** ^*a*^ (*n* = 1)	37.8	3.2	74.7	20.0	5.3
**4** ^*a*^ (*n* = 2)	31.2	4.2	62.3	30.0	7.7

^*a*^Group electronegativity values for calculation^22^ of ^3^*J*_HF_ were taken from the literature^23^ (**2**: 0.76 [SH group] and **3**/**4**: 0.69 [SO_2_Cl group]).

## DFT conformational analysis

**Fig. 3:**
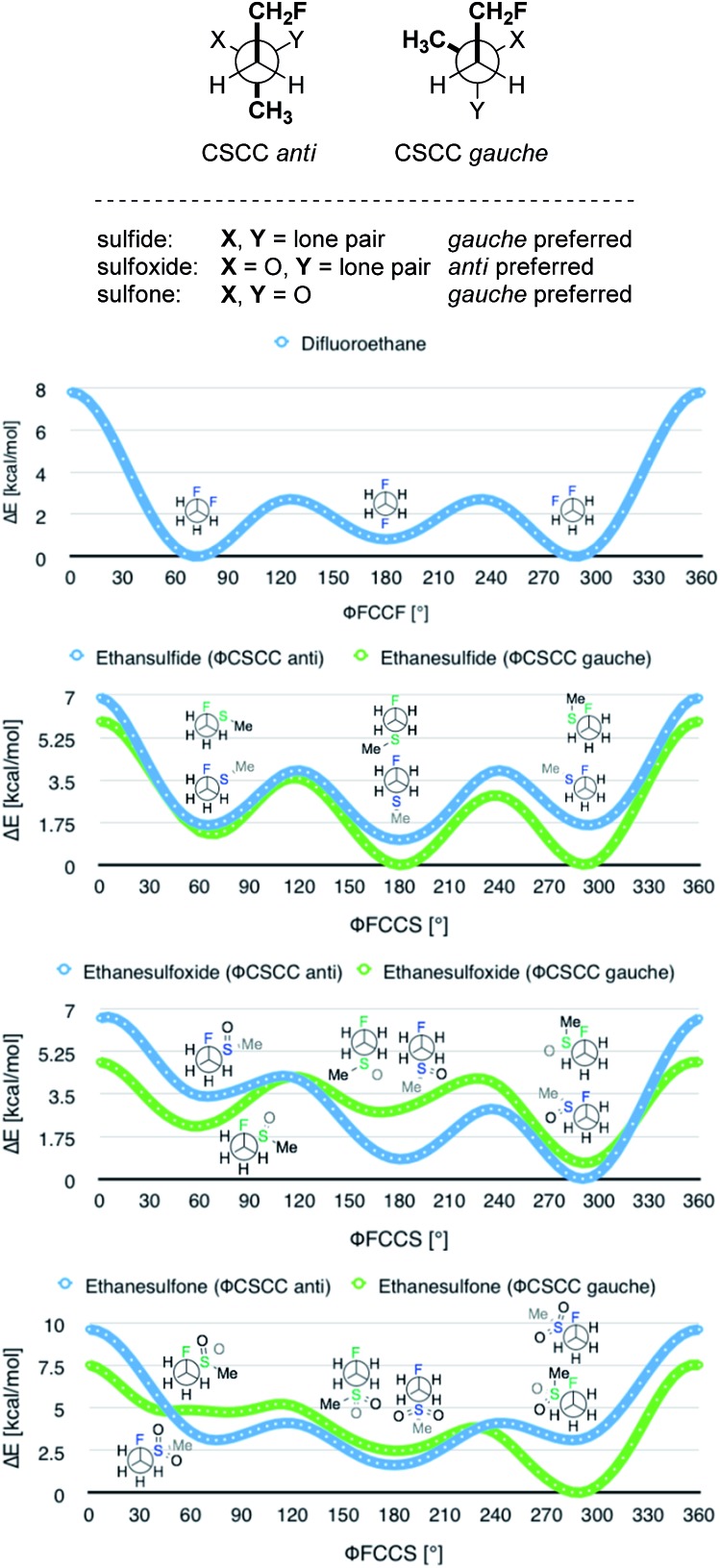
Conformational preferences around the CSCC bond for 1,2-difluoroethane (**8**), (2-fluoroethyl)(methyl)-sulfide (**9**), -sulfoxide (**10**), and -sulfone (**11**). Results obtained with B3LYP/6-311+G(d,p) in vacuum.

In order to quantify the observed conformational preferences using DFT, a series of structures containing the key F–C–C–S(O)_*n*_ unit (*n* = 0, 1 and 2) were optimised at the B3LYP^24^/6-311+G(d,p)^25^ level of theory. Solvation by dichloromethane was taken into account using the integral equation formalism polarizable continuum model (IEFPCM).^26^ Dichloromethane was chosen to ensure consistency with the NMR solution phase conformational analysis. The choice of basis set was based on a previous computational study of the *gauche* effect in α-X-β-fluoro-ethane derivatives (X = F, NR, OR, CR) by O'Hagan and co-workers.4*c* All computations were performed using Gaussian09.^27^ Free energy corrections were calculated using Truhlar's *quasi*-harmonic approximation.^28^ The lowest energy conformers of 1,2-difluoroethane (**8**) and the corresponding (2-fluoroethyl)-(methyl)-derivatives (sulfide = **9**, sulfoxide = **10**, sulfone = **11**) were investigated (Table 3). Additionally, the C_α_–C_β_ bond rotational profiles (step size = 5°, 72 steps, B3LYP/6-311+G(d,p) in vacuum) of **9–11**, with both the CSCC *anti* and *gauche* conformations, were calculated and compared to the rotational profile of 1,2-difluoroethane (**8**) (Fig. 3, top).

**Table 3:** Calculated relative conformational energies and torsion angle of 1,2-difluoroethane (**8**), (2-fluoroethyl)(methyl)-sulfide (**9**), -sulfoxide (**10**), and -sulfone (**11**). Results obtained with B3LYP/6-311+G(d,p)/IEFPCM. Only the *gauche* conformer is shown for simplicity

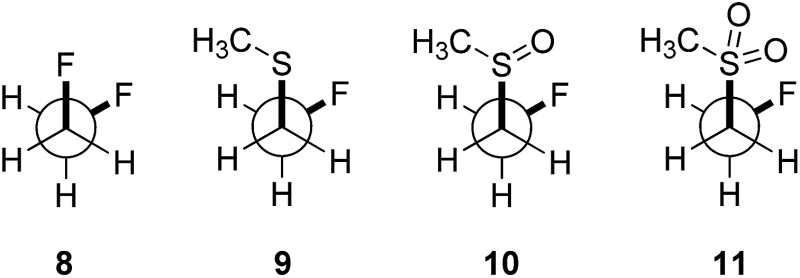						
	*φ*_CSCC_	*φ*_FCC(F/S)_	Δ*G* [kcal mol^–1^]	*φ*_FCC(F/S)_ [°]	*Q*_S_ ^*a*^	*μ* ^*b*^ [D]
**8**	**—**	**–*gauche***	**0.0**	**69**	**—**	**3.71**
	—	*anti*	1.9	180	—	0.00
**9**	*anti*	–*gauche*	1.0	–66	0.01	4.11
	*anti*	*anti*	1.2	180	0.01	2.30
	–*gauche*	–*gauche*	0.7	66	0.23	4.08
	**+*gauche***	***anti***	**0.0**	**–179**	**0.23**	**2.20**
	+*gauche*	–*gauche*	0.5	–68	0.21	1.89
**10**	*anti*	+*gauche*	1.8	67	0.67	7.97
	*anti*	*anti*	1.0	180	0.68	5.03
	***anti***	**–*gauche***	**0.0**	**–67**	**0.70**	**5.98**
	+*gauche*	+*gauche*	1.4	62	0.64	6.04
	+*gauche*	*anti*	2.1	170	0.66	5.20
	+*gauche*	–*gauche*	0.9	–70	0.66	4.44
	–*gauche*	*anti*	1.9	–166	0.65	5.38
**11**	*anti*	–*gauche*	1.2	–70	0.57	8.01
	*anti*	*anti*	1.6	180.0	0.51	5.15
	**+*gauche***	**–*gauche***	**0.0**	**–70**	**0.45**	**4.91**
	–*gauche*	*anti*	1.8	–178	0.42	5.26
	–*gauche*	–*gauche*	2.1	–76	0.39	7.79

^*a*^Mulliken atomic charges with hydrogens summed into heavy atoms.

^*b*^Molecular dipole moment in Debye.

This analysis confirmed that the well known *gauche* preference of 1,2-difluoroethane (**8**) is also inherent to the linear sulfoxide and sulfone derivatives (Δ*G*_*anti*/*gauche*_ = 1.9, 1.0, and 1.6 kcal mol^–1^, for **8**, **10** and **11** respectively). However, the sulfide derivative **9** displays a slight preference for the *anti* conformation (Δ*G*_*gauche*/*anti*_ = 0.5 kcal mol^–1^); this is at variance with the X-ray structures of **2** (Fig. 2). As expected, the *gauche* conformation appears to be more pronounced in structures bearing a more electropositive vicinal sulfur atom (S^+^–O^–^, SO_2_).

As a quantitative measurement for this effect, the Mulliken atomic charges (*Q*_S_) of the sulfur atom in each of the conformers studied were calculated. These are listed in Table 3 (right). Interestingly, comparison of the energy *minima* in compounds **9–11** displayed some variation with respect to the CSCC chain. Whereas the sulfide **9** and the sulfone **11** position the two alkyl-groups *gauche* to each other in the lowest lying minima, the sulfoxide **10** preferentially orients the groups *anti* (Fig. 3).

A computational analysis of the linear 4-nitrothiophenol derived systems **5–7** (Table 4) revealed similar trends to those observed with the ethane derivatives (Table 3, also see Scheme 2). The sulfide derivative **5** displayed no significant preference for the *gauche* or *anti* conformation (**5**, Δ*G*_*gauche*/*anti*_ = 0.1 kcal mol^–1^), whilst the sulfoxide and sulfone derivatives both exhibited a *gauche* preference (**6** and **7**, Δ*G*_*anti*/*anti*_ = 1.3 and 1.3 kcal mol^–1^, respectively).

**Table 4:** Calculated relative conformational energies and torsion angle of (2-fluoroethyl) (4-nitrophenyl)-sulfide (**5**), -sulfoxide (**6**), and -sulfone (**7**). Results obtained with B3LYP/6-311+G(d,p)/IEFPCM. Only the *gauche* conformer is shown for simplicity

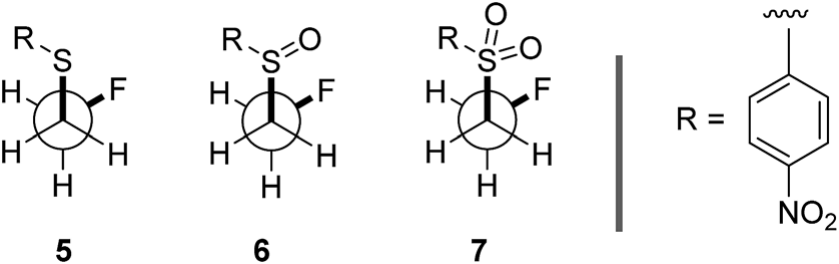						
	*φ*_CSCC_	*φ*_FCCS_	Δ*G* [kcal mol^–1^]	*φ*_FCCS_ [°]	*Q*_S_ ^*a*^	*μ* ^*b*^ [D]
**5**	***anti***	**+*gauche***	**0.0**	**66**	**–0.67**	**7.07**
	*anti*	*anti*	0.4	180	–0.65	6.20
	+*gauche*	+*gauche*	0.2	64	–0.38	6.68
	+*gauche*	*anti*	0.1	179	–0.34	6.12
	+*gauche*/*eclipsed*	–*gauche*	0.1	–69	–0.30	9.55
**6**	*anti*	+*gauche*	1.7	68	0.85	6.62
	*anti*	*anti*	1.3	180	0.95	4.32
	***anti***	**–*gauche***	**0.0**	**–66**	**0.89**	**4.65**
	+*gauche*	+*gauche*	1.7	62	1.01	4.62
	+*gauche*	*anti*	2.9	173	1.01	4.15
	*eclipsed*	–*gauche*	1.8	–67	0.82	6.55
**7**	*anti*	–*gauche*	0.9	–70	0.63	5.74
	*anti*	*anti*	1.3	–179	0.58	2.50
	**+*gauche***	**–*gauche***	**0.0**	**–72**	**0.66**	**5.85**
	+*gauche*	*anti*	1.4	179	0.63	2.55
	+*gauche*	+*gauche*	1.4	72	0.61	5.65

^*a*^Mulliken atomic charges with hydrogens summed into heavy atoms.

^*b*^Molecular dipole moment in Debye.

The three possible rotamers of cyclic compounds **2**, **3** and **4** were also investigated (Table 5). For the sulfoxide derivative **3** both diastereoisomers (oxygen *anti***3a**, and *syn***3b**) were considered, although only the *anti* diastereoisomer was isolated following oxidation. In this case, the *syn* conformer is significantly higher in energy than the *anti* (Δ*G*_*syn*/*anti*_ = 3.7 kcal mol^–1^).

**Table 5:** Calculated relative conformational energies and torsion angle of cyclic sulfide **2**, sulfoxide **3**, and sulfone **4**. Results obtained with B3LYP/6-311+G(d,p)/IEFPCM. Only the *gauche* conformer is shown for simplicity

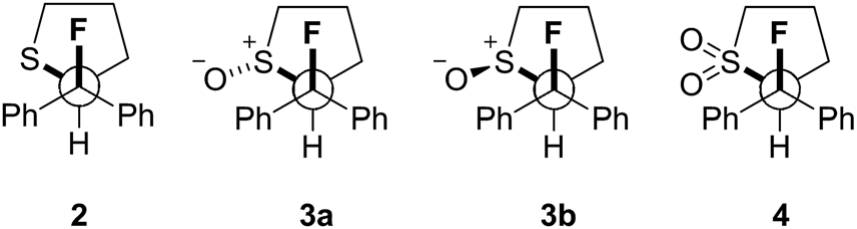						
	Δ*d*_S1–C4/S1–C1_ [Å]	*φ*_FCCS_	Δ*G* [kcal mol^–1^]	*φ*_FCCS_ [°]	*Q*_S_ ^*a*^	*μ* ^*b*^ [D]
**2**	0.021	+*gauche*	3.3	64	0.07	4.92
	0.018	*anti*	1.7	–176	–0.08	1.76
	**0.022**	**–*gauche***	**0.0**	**–63**	–0.12	3.25
**3a** ^*c*^	0.057	+*gauche*	2.4	46	0.84	8.38
	0.045	*anti*	2.7	172	0.84	5.51
	**0.050**	**–*gauche***	**0.0**	**–63**	0.81	5.09
**3b** ^*d*^	0.012	+*gauche*/*eclipsed*	5.7	26	0.78	7.15
	0.012	*anti*	4.2	158	0.62	3.89
	0.042	–*gauche*	3.7	–70	0.68	7.25
**4**	0.039	+*gauche*	3.0	34	0.71	9.12
	0.034	*anti*	2.9	161	0.52	5.42
	**0.050**	**–*gauche***	**0.0**	**–66**	0.70	6.88

^*a*^Mulliken atomic charges with hydrogens summed into heavy atoms.

^*b*^Molecular dipole moment in Debye.

^*c*^Oxygen *anti*.

^*d*^Oxygen *syn*.

**Fig. 4:**
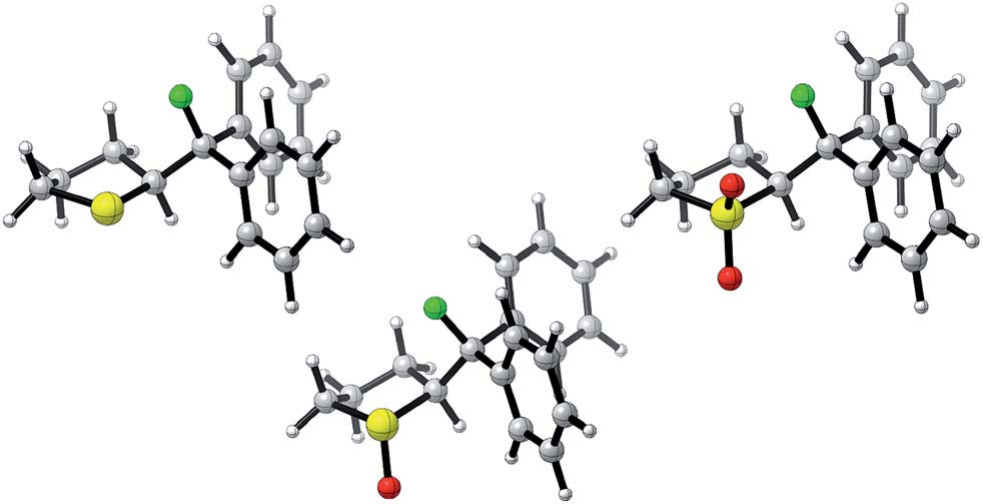
Global energy *minima* for cyclic sulfide **2**, sulfoxide **3**, and sulfone **4**, all adopting the experimentally observed *synclinal-endo* (*gauche*) conformation. Results obtained with B3LYP/6-311+G(d,p)/IEFPCM. S = yellow, O = red, F = green.

The sulfide (**2**) again shows the smallest energetic difference between the lowest lying *anti* and *syn* conformations, consistent with the results for the acyclic derivatives. Contrary to the other structures investigated, this cyclic derivative exhibits a significant preference for the *gauche* conformation (Δ*G*_*anti*/*gauche*_ = 1.7 kcal mol^–1^). For the derivatives bearing a more electron deficient sulfur atom, the *gauche* preference is more pronounced (**3** and **4**, Δ*G*_*anti*/*gauche*_ = 2.4 and 2.9 kcal mol^–1^, respectively). The *synclinal-endo* conformation is significantly lower in energy than the *synclinal-exo* conformation, and the latter is higher in energy than the *anti*. The optimised structures displayed the same lengthening of the S1–C4 bond as was observed by crystallography. The global energy minima for compounds **2**, **3** and **4** are shown in Fig. 4 and closely match the corresponding X-ray structures (Fig. 2).

## Conclusions

Computation and experiment confirm that the F–C–C–S motif has an intrinsic *gauche* conformational preference. Conformational analysis in both solid and solution phase confirmed that the C–F bond aligns *anti* to a vicinal C–H bond, reasonably to allow for stabilising hyperconjugative interactions of the type *σ*_C–H_ → σ*C–F. The Δ*G*_*gauche*/*anti*_ value is larger when the sulfur centre is more electron deficient; this is especially pronounced in sulfoxides. Computational analyses indicate that the conformational preference is not solely due to overall molecular dipole minimisation (Table 3, 4 and 5, right column). This study extends the well-known *gauche* effect to 3^rd^ Period substituents, and validates the notion that the F–C–C–S(O)_*n*_ unit may be strategically embedded into functional scaffolds to achieve acyclic conformational control.

## Supplementary Material

Supplementary information

Crystal structure data
