# Supplementary material for: Can acyclic conformational control be achieved via a sulfur–fluorine gauche effect?
Source: Chem Sci. 2015 Apr 17;6(6):3565–71. doi: 10.1039/c5sc00871a (PMC5659174; doi:10.1039/c5sc00871a)
Supplement: Supplementary file 1 [file SC-006-C5SC00871A-s001.pdf]

Electronic Supplementary Material (ESI) for Chemical Science.
This journal is © The Royal Society of Chemistry 2015

- General Information S2
- Experimental Section S2
- NMR Spectra of Key Compounds S12
- X-Ray Data of Key Compounds S18
- Computational Data S20
- References S56

General Information

All chemicals were purchased as reagent grade and used without further purification except for 4-nitrothiophenol which was purchased as technical grade (80%) and purified by recrystallization from boiling EtOH. Anhydrous CH_2Cl_2 and THF were obtained by passing them over a column packed with Al_2O_3 (pellets, 3 mm) under an atmosphere of Argon. Solvents for extractions and chromatographic purifications were bought as technical grade and distilled on the rotary evaporator prior to use. Analytical thin layer chromatography was performed on glass plates pre-coated with SiO_2-60 F_{254} (Merck) and visualised with UV-light (254 nm) or with $KMnO_4$ or CAM solution. Column chromatography was performed on SiO_2-60 (230-400 mesh ASTM, Fluka). NMR spectra were recorded at room temperature on a *Bruker AV300*, *AV400* or an *Agilent DD2 600* by the NMR service of the Organisch-Chemisches Institut, Westfälische Wilhelms-Universität Münster. The chemical shifts *δ* are reported in ppm and referenced to the residual solvent peak as internal standard. The multiplicity is abbreviated as: s (singlet), d (doublet), t (triplet), q (quadruplet), m (multiplet) and br (broad). The coupling constant *J* is given in Hz. Melting points were measured on a *Büchi B-545* melting-point apparatus utilising open glass capillaries and are uncorrected. IR spectra were recorded on a *Perkin-Elmer 100 FT-IR* spectrometer and are reported in cm^{-1} with the intensities of the bands assigned: w (weak), m (medium) and s (strong). Optical rotations were measured on a *JASCO P2000* polarimeter. HPLC spectra were recorded on an *Agilent 1100 series* or *Agilent 1260 Infinity* using a Chiracel OJ column (5 µm, 250 x 4.6 mm) or Reprosil Chiral AM column (10 µl, 240 x 4.6 mm) and *n*-hexanes/*i*-PrOH as eluent. High resolution mass spectra (HRMS) were measured by the MS service of the Organisch-Chemisches Institut, Westfälische Wilhelms-Universität Münster.

Experimental Section

5-Bromo-1,1-diphenylpentan-1-ol (S1)[1]

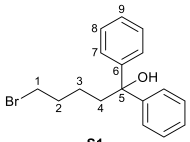

S1

A flame-dried two-neck flask was charged with magnesium turnings (1.704 g, 70.1 mmol, 2.9 equiv.) and a catalytic amount of iodine and purged with argon. Dry THF (50 ml) and phenyl bromide (1.0 ml, 9.5 mmol). were added sequentially and the resulting mixture was stirred for 10 min. The rest of phenyl bromide (6.6 ml, 62.7 mmol) was added in a drop wise fashion over 30 min. The resulting grey mixture was stirred for further 30 min, prior to the addition of a solution of ethyl 5-bromovalerate (3.8 ml, 24.0 mmol, 1.0 equiv.) in THF (20 ml) at 0 °C

within 15 min. After stirring over night at room temperature, the mixture was quenched with sat. aq. NH_4Cl (10 ml) at 0 °C. The aqueous layer was separated and extracted with EtOAc (3 x 40 ml). The combined organic layers were washed with sat. aq. $NaHCO_3$ (2 x 20 ml), brine (25 ml), dried over $NaSO_4$, and concentrated under reduced pressure to give a brown oil, which solidified upon storage in the fridge. The resulting residue was recrystallised from boiling hexanes/CH_2Cl_2 (v/v 4:1) yielding the desired product as off-white crystals (6.26 g, 82%). An analytical pure sample was obtained via column chromatography (silica, Cy/EtOAc 10:1).

R_f = 0.26 (Cy/EtOAc 10:1); m.p. = 81.8−83.1 °C; ^{1}H NMR (400 MHz, $CDCl_3$) δ = 7.43−7.39 (4H, m, H−C7), 7.34−7.28 (4H, m, H−C8), 7.27−7.24 (2H, m, H−C9), 3.39 (2H, t, J = 6.9, H−C1), 2.34−2.27 (2H, m, H−C4), 2.09 (1H, s, H−O), 1.89 (2H, p, J = 7.1, H−C2), 1.50−1.38 (2H, m, H−C3); ^{13}C NMR (101 MHz, $CDCl_3$) δ = 146.9 (2C, C6), 128.3 (4C, C8), 127.1 (2C, C9), 126.1 (4C, C7), 78.2 (C5), 41.2 (C4), 33.6 (C1), 33.2 (C2), 22.8 (C3); IR (ATR) $\tilde{\nu}$ = 3525m, 3027w, 2937m, 2867w, 2321w, 1957w, 1598w, 1489m, 1469w, 1447m, 1392w, 1355w, 1301w, 1265m, 1233w, 1209w, 1189m, 1172m, 1134m, 1059m, 1046w, 1031m, 1002w, 959m, 912m, 897m, 871w, 856m, 774m, 751m, 731m, 713m, 695s cm^{-1}; HRMS (ESI): m/z: 341.0525 ($[M+Na]^+$, calcd. for $C_{17}H_{19}BrONa^+$: 341.0511). Analytical data in agreement with the literature.[1]

(5-Bromopent-1-ene-1,1-diyl)dibenzene (S2)[1]

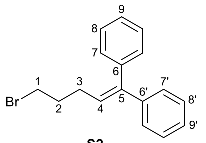

S2

A solution of **S1** (6.48 g, 21.1 mmol, 1.0 equiv.) and TsOH•H_2O (386 mg, 2.1 mmol, 0.1 equiv.) in toluene (85 ml) was heated to 75 °C for 3.5 h. After cooling to rt, the mixture was diluted with H_2O (15 ml) and the aqueous layer was extracted with EtOAc (3 x 10 ml). The combined organic layers were dried over Na_2SO_4 and concentrated under reduced pressure to give a brown oil. Purification via column chromatography (silica, Cy/EtOAc 97:3) afforded the desired product as a slightly yellow oil (6.13 g, 96%).

R_f = 0.29 (Cy); ^{1}H NMR (600 MHz, $CDCl_3$) δ = 7.39 (2H, m, H−C8/8'), 7.33 (1H, m, H−C7/7'), 7.27 (2H, m, H−C8/8'), 7.23 (3H, m, H−C7/7'), 7.18 (2H, m, H−C9/9'), 6.05 (1H, t, J = 7.5, H−C4), 3.39 (2H, t, J = 6.9, H−C1), 2.28 (2H, q, J = 7.4, H−C3), 2.01 (2H, m, H−C2); ^{13}C NMR (151 MHz, $CDCl_3$) δ = 143.2 (C6/6'), 142.6 (C6/6'), 140.0 (C5), 129.9 (2C, C9/9'), 128.4 (2C, C8/8'), 128.3 (2C, C8/8'), 127.7 (C4), 127.3 (2C, C7/7'), 127.2 (2C, C7/7'), 33.2 (C2), 33.2 (C1), 28.6 (C3); IR (ATR) $\tilde{\nu}$ = 3079w, 3054w, 3021w, 2960w, 2118w, 1597w,

1575w, 1494m, 1443m, 1364w, 1244w, 1202w, 1156w, 1073m, 1030w, 1001w, 923w, 896w, 869w, 760m, 700m cm^{-1}; HRMS (APCI): *m/z*: 300.0506 ($[M]^+$, calcd. for $C_{17}H_{17}Br^+$: 300.0508). Analytical data in agreement with the literature.[1]

(*R*)-3-(3-Bromopropyl)-2,2-diphenyloxirane (S3)[1]

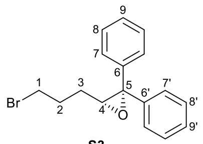

S3

To a solution of **S2** (3.839 g, 12.7 mmol, 1.00 equiv.) in CH_3CN/DMM (v/v 1:2, 96 ml) was added a solution of $Na_2B_4O_7$•10H_2O (0.1 M in Na_2EDTA 4 x 10^{-4} M, 64 ml) followed by the addition of Bu_4NHSO_4 (184 mg, 0.5 mmol, 0.04 equiv.) and Shi catalysator **S6** (1.650 g, 6.4 mmol, 0.50 equiv.). The mixture was cooled to 0 °C and a solution of oxone in $Na_2EDTA_{(aq)}$ (4 x 10^{-4} M, 80 ml) and an aq. solution of K_2CO_3 (19.64 g, 142.1 mmol, 11.15 equiv. in 80 ml H_2O) were added simultaneously in a dropwise fashion over 3.5 h. The mixture was stirred for additional 4 h at 0 °C and then quenched by the addition of H_2O (125 ml). The reaction mixture was extracted with Cy (2 x 200 ml) and the combined organic layers were dried over Na_2SO_4, and concentrated under reduced pressure to give a slightly yellow oil. Purification via column chromatography (silica, Cy/EtOAc 40:1 → 30:1) afforded the desired product as colourless oil (2.727 g, 68%, 94% *ee*).

R_f = 0.32 (Cy/EtOAc 40:1); $[\alpha]_D^{25}$: +35.1° (*c* = 1.0, $CHCl_3$; [lit. $[\alpha]_D^{28}$: +37.0°, *c* = 1.0, $CHCl_3$]); ^1H NMR (300 MHz, $CDCl_3$) δ = 7.43−7.36 (4H, m, H−C7), 7.35−7.26 (6H, m, H−C8/9), 3.47−3.31 (3H, m, H−C3/4), 2.18−1.93 (2H, m, H−C1), 1.78−1.65 (1H, m, H−C2), 1.41−1.26 (1H, m, H−C2); ^{13}C NMR (75 MHz, $CDCl_3$) δ = 140.9 (C6/6'), 137.4 (C6/6'), 128.5 (C7/7'), 128.3 (C8/8'), 128.1 (C7/7'), 128.0 (C9/9'), 127.9 (C9/9'), 127.1 (C8/8'), 65.6 (C4), 33.1 (C3), 29.8 (C1), 28.4 (C2); IR (ATR) $\tilde{\nu}$ = 3060w, 3028w, 2964w, 1602w, 1495m, 1447m, 1277w, 1249m, 1208w, 1156w, 1078w, 1030w, 1002w, 932m, 907m, 891m, 845w, 816w, 763m, 701m cm^{-1}; HRMS (ESI): *m/z*: 339.0354 ($[M+Na]^+$, calcd. for $C_{17}H_{17}BrONa^+$: 339.0355); enantioselectivity was determined by HPLC analysis (Chiracel-OJ column, 1.0 ml/min, 220 nm, 20% *i*-PrOH/hexanes: retention time: 17.0 min (minor) and 29.4 min (major)). Analytical data in agreement with the literature.[1]

(*S*)-Diphenyl(tetrahydrothiophen-2-yl)methanol (1)[1]

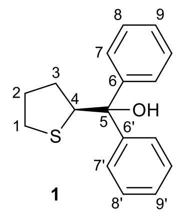

A suspension of **S3** (3.234 g, 10.2 mmol, 1.0 equiv.) and $Na_2S \cdot 3H_2O$ (2.710 g, 20.4 mmol, 2.0 equiv.) in EtOH (40 ml) was sonicated at rt for 37.5 h. The solvent was removed under reduced pressure and the resulting residue was dissolved in H_2O (100 ml) and CH_2Cl_2 (80 ml). The aqueous layer was extracted with CH_2Cl_2 (3 x 80 ml) and the combined organic layers were washed with brine, dried over Na_2SO_4, and concentrated under reduced pressure to give a yellow oil which solidified upon standing. The residue was recrystallised from boiling hexanes affording the product as a white crystalline solid (2.060 g, 75%, 99% *ee*).

m.p. = 72−75 °C; $[\alpha]_D^{28}$: −93.4° (*c* = 1.0, $CHCl_3$; [lit. $[\alpha]_D^{28}$: −120.4°, *c* = 1.0, $CHCl_3$]); ^{1}H NMR (400 MHz, CD_2Cl_2) δ = 7.57−7.53 (2H, m, H−C7), 7.49−7.45 (2H, m, H−C7’), 7.34−7.25 (4H, m, H−C8/8’), 7.24−7.15 (2H, m, H−C9/9’), 4.73 (1H, dd, *J* = 8.7, 6.6, H−C4), 3.59 (1H, s, H−O), 2.88−2.83 (2H, m, H−C1), 2.22−2.15 (1H, m, H−C2), 1.90−1.72 (2H, m, H−C2/3), 1.67−1.60 (1H, m H−C2); ^{13}C NMR (101 MHz, CD_2Cl_2) δ = 148.9 (C6’), 145.7 (C6), 128.7 (C8/8’), 128.6 (C8/8’), 127.6 (C9’), 127.1 (C9), 126.5 (C7), 125.9 (C7’), 78.3 (C5), 59.9 (C4), 34.0 (C1), 32.4 (C2), 32.3 (C3); IR (ATR) $\tilde{\nu}$ = 3473w, 3088w, 3051w, 3037w, 2962w, 2925w, 2899w, 2856w, 2287w, 2163w, 2079w, 2012w, 1955w, 1887w, 1805w, 1597w, 1490m, 1458w, 1444m, 1392w, 1358w, 1329m, 1308w, 1296w, 1263w, 1245w, 1219w, 1193m, 1167m, 1108w, 1064m, 1049w, 1031w, 1001w, 985m, 976m, 907w, 888m, 882m, 857w, 784m, 754m, 738m, 696s, 650m cm^{-1}; HRMS (NSI): *m/z*: 293.0968 ($[M+Na]^+$, calcd. for $C_{17}H_{18}OSNa^+$: 293.0970); enantioselectivity was determined by HPLC analysis (Reprosil Chiral-AM column, 1.0 ml/min, 220 nm, 5% *i*-PrOH/hexanes: retention time: 5.5 min (minor) and 7.3 min (major)). Analytical data in agreement with the literature.[1]

(*S*)-2-(Fluorodiphenylmethyl)tetrahydrothiophene (2)

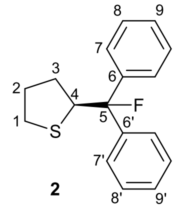

A flame-dried flask was charged with **1** (136 mg, 0.5 mmol, 1.0 equiv.) and Na_2CO_3 (213 mg, 2.0 mmol, 4.0 equiv., dried under hv at 600 °C), purged with argon and dry CH_2Cl_2 (6 ml) was added. The suspension was cooled to 0 °C and DAST (270 µl, 2.0 mmol, 4.0 equiv.) was added slowly. After stirring for 16 h while warming to rt, the reaction mixture was quenched by the careful addition of aq. sat. $NaHCO_3$ (5 ml), resulting in vigorous gas evolution. The aqueous phase was extracted with CH_2Cl_2 (3 x 5 ml) and the combined organic layers were washed with brine, dried over Na_2SO_4, and concentrated under reduced pressure to give a brown oil

which solidified upon addition of a few drops of hexanes. Recrystallisation from boiling hexanes afforded the desired product as a white crystalline solid (73 mg, 54%).

m.p. = 81−82 °C; $[\alpha]_D^{23}$: +5.8° (*c* = 0.25, $CHCl_3$); ^{1}H NMR (400 MHz, CD_2Cl_2) δ = 7.51−7.49 (2H, m, H−C7), 7.45−7.40 (2H, m, H−C7'), 7.39−7.31 (4H, m, H−C8/8'), 7.31−7.22 (2H, m, H−C9/9'), 4.68−4.54 (1H, m, H−C4), 2.93−2.77 (2H, m, H−C1), 2.22−2.08 (1H, m, H−C1), 1.96−1.76 (3H, m, H−C2/3); ^{13}C NMR (101 MHz, CD_2Cl_2) δ = 144.3 (d, $^2J_{CF}$ = 23.4, C6'), 143.85 (d, $^2J_{CF}$ = 24.9, C6), 128.9 (d, $^4J_{CF}$ = 1.4, C8/8'), 128.8 (d, $^4J_{CF}$ = 1.0, C8/8'), 128.2 (d, $^5J_{CF}$ = 1.4, C9/9'), 128.0 (d, $^5J_{CF}$ = 1.2, C9/9'), 125.7 (d, $^3J_{CF}$ = 8.7, C7), 125.3 (d, $^3J_{CF}$ = 9.3, C7'), 100.0 (d, $^1J_{CF}$ = 184.7, C5), 56.8 (d, $^2J_{CF}$ = 23.4, C4), 33.8 (C1), 32.0 (d, $^4J_{CF}$ = 0.6, C2) 31.9 (d, $^3J_{CF}$ = 3.2, C3); ^{19}F NMR (282 MHz, CD_2Cl_2) δ = −164.05 (1F, d, $^3J_{HF}$ = 29.1, F−C5); IR (ATR) $\tilde{\nu}$ = 3056w, 3026w, 2982w, 2963w, 2949w, 2933w, 2908w, 2864w, 2325w, 2162w, 2064w, 1974w, 1955w, 1901w, 1831w, 1806w, 1597w, 1584w, 1490m, 1448m, 1439m, 1338w, 1322w, 1288w, 1266w, 1250w, 1228m, 1195m, 1187m, 1165w, 1126w, 1114w, 1066m, 1050w, 1033w, 1020w, 1001w, 972m, 936m, 923w, 912w, 895m, 876m, 854m, 787m, 757m, 740s, 705m, 696s cm^{-1}; HRMS (APCI): *m/z*: 253.1038 ($[M-HF]^+$, calcd. for $C_{17}H_{17}S^+$: 253.1045).

(2*S*)-2-(Fluorodiphenylmethyl)tetrahydrothiophene 1-oxide (3)[2]

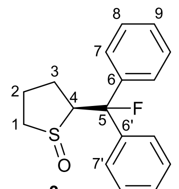

3

To a solution of **2** (68 mg, 0.25 mmol, 1.0 equiv.) in CH_2Cl_2 (1.25 ml) was added *m*CPBA (≤ 77%, 58 mg, 0.25 mmol, 1.0 equiv.) at 0 °C and the mixture was stirred for 50 h while warming to rt. The reation mixture was washed with aq. sat. $NaHCO_3$ (3 x 1 ml) and brine and the organic layer was dried over Na_2SO_4 and concentrated under reduced pressure to give an off-white viscous oil. Purification via column chromatography (silica, Cy/EtOAc 2:3 → 1:2 → 1:1) afforded the desired product as a white crystalline solid (49 mg, 68%) in a diastereomeric ratio of 97:3 (determined by ^{1}H and ^{19}F NMR).

R_f = 0.23 (Cy/EtOAc 1:2); m.p. = decomp. >136 °C; $[\alpha]_D^{23}$: −65.5° (*c* = 0.25, $CHCl_3$); ^{1}H NMR (400 MHz, CD_2Cl_2) δ = 7.61−7.57 (2H, m, H−C7'), 7.46−7.25 (8H, m, H−C7/8/8'/9/9'), 4.13 (1H, ddd, $^3J_{HF}$ = 38.2, J = 9.3, 8.1, H−C4), 3.02−2.95 (1H, m, H−C1), 2.64−2.45 (2H, m, H−C1/2), 2.39−2.31 (1H, dtd, J = 11.6, 5.1, 2.1, H−C2), 2.25−2.16 (1H, dddd, J = 13.0, 6.9, 3.2, 1.6, H−C3), 1.82−1.70 (1H, m, H−C3); ^{13}C NMR (101 MHz, CD_2Cl_2) δ = 143.1 (d, $^2J_{CF}$ = 23.6, C6), 142.5 (d, $^2J_{CF}$ = 24.2, C6'), 129.3 (d, $^5J_{CF}$ = 1.0, C9/9'), 129.2 (d, $^5J_{CF}$ = 1.6, C9/9'), 128.8 (d, $^4J_{CF}$ = 1.4, C8/8'), 128.4 (d, $^4J_{CF}$ = 1.0, C8/8'), 125.4 (d, $^3J_{CF}$ = 8.8, C7'),

124.9 (d, $^3J_{CF}$ = 9.4, C7), 99.8 (d, $^1J_{CF}$ = 178.9, C5), 81.6 (d, $^2J_{CF}$ = 22.1, C4), 54.3 (C1), 29.2 (d, $^3J_{CF}$ = 3.2, C3), 27.5 (C2); ^{19}F NMR (282 MHz, CD_2Cl_2) δ = −160.9 (1F, d, $^3J_{HF}$ = 38.2, F−C5); IR (ATR) $\tilde{\nu}$ = 3083w, 3055w, 3023w, 2956w, 2936w, 2870w, 1600w, 1584w, 1491w, 1451m, 1442m, 1416w, 1337w, 1320w, 1286m, 1249w, 1231w, 1208w, 1182w, 1167w, 1152w, 1108w, 1066w, 1049m, 1022m, 994m, 975m, 961m, 922w, 909m, 893m, 874w, 853w, 787m, 758m, 735m, 699s, 653m cm^{-1}; HRMS (ESI): *m/z*: 311.0889 ([M+Na]$^+$, calcd. for $C_{17}H_{17}FSONa^+$: 311.0876). NMR data given for the major diastereomer, remaining analytical data given for the diastereomeric mixture.

(2*S*)-2-(Fluorodiphenylmethyl)tetrahydrothiophene 1,1-dioxide (4)[2]

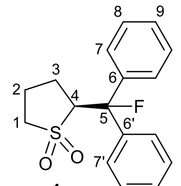

4

To a solution of **2** (29 mg, 0.11 mmol, 1.0 equiv.) in CH_2Cl_2 (0.5 ml) was added *m*CPBA (≤ 77%, 73 mg, 0.32 mmol, 3.0 equiv.) at 0 °C and the mixture was stirred for 17 h while warming to rt. The reaction mixture was diluted with CH_2Cl_2 (3 ml) and washed with aq. sat. $NaHCO_3$ (3 x 1 ml). The organic layer was dried over Na_2SO_4 and concentrated under reduced pressure to give an off-white solid. Purification via column chromatography (silica, Cy/EtOAc 3:1) afforded the desired product as a white solid (27 mg, 85%).

R_f = 0.39 (Cy/EtOAc 3:1); m.p. = 132.0−133.2 °C; $[\alpha]_D^{28}$: −82.6° (*c* = 0.2, $CHCl_3$); ^{1}H NMR (600 MHz, CD_2Cl_2) δ = 7.57 (2H, dt, *J* = 8.3, 1.8, H−C7'), 7.42−7.30 (7H, m, H−C7/8/8'/9'), 7.27 (1H, tt, *J* = 6.3, 1.7, H−C9), 4.27 (1H, dt, $^3J_{HF}$ = 31.4, *J* = 8.9, H−C4), 3.15−3.09 (1H, m, H−C1), 3.02−2.95 (1H, m, H−C1), 2.25−2.14 (2H, m, H−C2/3), 2.09−1.99 (2H, m, H−C2/3); ^{13}C NMR (151 MHz, CD_2Cl_2) δ = 142.9 (d, $^2J_{CF}$ = 24.7, C6'), 141.6 (d, $^2J_{CF}$ = 23.3, C6), 129.3 (d, $^4J_{CF}$ = 1.9, C8), 129.0 (d, $^4J_{CF}$ = 1.1, C8'), 128.6 (d, $^5J_{CF}$ = 1.4, C9'), 128.4 (d, $^5J_{CF}$ = 1.1, C9), 125.3 (d, $^3J_{CF}$ = 8.5, C7'), 124.6 (d, $^3J_{CF}$ = 9.9, C7), 98.0 (d, $^1J_{CF}$ = 187.2, C5), 67.0 (d, $^2J_{CF}$ = 22.5, C4), 53.7 (C1), 26.4 (d, $^3J_{CF}$ = 4.2, C3), 20.1 (C2); ^{19}F NMR (564 MHz, CD_2Cl_2) δ = −169.3 (1F, d, $^3J_{HF}$ = 31.5, F−C5); IR (ATR) $\tilde{\nu}$ = 2969w, 2945w, 2165w, 1981w, 1598w, 1492w, 1450m, 1418w, 1312m, 1265m, 1249m, 1229w, 1206.6w, 1182w, 1163m, 1129m, 1096m, 1068m, 1044m, 1031w, 1007w, 974m, 924w, 907w, 870m, 841w, 794m, 755m, 744m, 719m, 695s, 656m cm^{-1}; HRMS (ESI): *m/z*: 327.0826 ([M+Na]$^+$, calcd. for $C_{17}H_{17}FSO_2Na^+$: 327.0825).

2-Fluoroethyl 4-methylbenzenesulfonate (S4)[3]

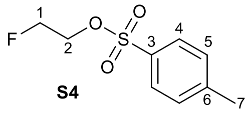

S4

To a solution of 2-fluoroethanol (590 µl, 10.0 mmol, 1.0 equiv.) in CH_2Cl_2 (15 ml) were added tosyl chloride (2.86 g, 15.0 mmol, 1.5 equiv.), Et_3N (2.1 ml, 15.0 mmol, 1.5 equiv.) and DMAP (122 mg, 1.0 mmol, 0.1 equiv.) successively. The mixture was stirred under an atmosphere of argon at rt for 20 h. The resulting organe-brown suspension was washed with 1 N HCl (10 ml), aq. sat. $NaHCO_3$ (15 ml), brine, dried over Na_2SO_4, and concentrated under reduced pressure to give a brown liquid. Purification of the crude product via column chromatography (silica, Cy/EtOAc 3:1) afforded the desired compound as a slightly yellow oil (1.87 g, 86%).

R_f = 0.33 (Cy/EtOAc 3:1); ^{1}H NMR (400 MHz, $CDCl_3$) δ = 7.82−7.78 (2H, m, H−C4), 7.38−7.33 (2H, m, H−C5), 4.64−4.61 (1H, m, H−C1), 4.52−4.49 (1H, m, H−C1), 4.31−4.27 (1H, m, H−C2), 4.24−4.21 (1H, m, H−C2), 2.45 (3H, s, H−C7); ^{13}C NMR (101 MHz, $CDCl_3$) δ = 145.3 (C6), 132.7 (C3), 130.1 (2C, C5), 128.1 (2C, C4), 80.7 (d, $^1J_{CF}$ = 174, C1), 68.6 (d, $^2J_{CF}$ = 21, C2), 21.8 (C7); ^{19}F NMR (282 MHz, $CDCl_3$) δ = −224.7 (1F, tt, $^2J_{HF}$ = 47.1, $^3J_{HF}$ = 27.2, F−C1); IR (ATR) $\tilde{\nu}$ = 3471w, 3052w, 2961w, 2925w, 2901w, 2856w, 1598w, 1494w, 1447m, 1402w, 1356m, 1303w, 1294w, 1263w, 1220w, 1190m, 1172m, 1109w, 1096m, 1064m, 1020m, 986m, 913m, 890m, 815m, 754m, 740m, 697m, 661m cm^{-1}; HRMS (ESI): *m/z*: 241.0306 ($[M+Na]^+$, calcd. for $C_9H_{11}FO_3SNa^+$: 241.0305). Analytical data in agreement with the literature.[2]

(2-Fluoroethyl)(4-nitrophenyl)sulfane (5)[4]

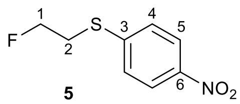

5

To a solution of **S4** (175 mg, 0.8 mmol, 1.0 equiv.) in toluene (3.2 ml) were added 4-nitrothiophenol (186 mg, 0.96 mmol, 1.2 equiv.) and DBU (145 µl, 0.96 mmol, 1.2 equiv.) resulting in a dark red mixture. After stirring for 16 h at rt, the reaction was quenched by the addition of aq. sat. NH_4Cl (3 ml) and diluted with H_2O and CH_2Cl_2. The aqueous layer was extracted with CH_2Cl_2 (3 x 10 ml) and the combined organic layers were washed with brine, dried over Na_2SO_4, and concentrated under reduced pressure to give a yellow suspension. The suspension was filtered over a plug of cotton using Et_2O removing most of the solid impurity. Subsequent purification via column chromatography (silica, pentane/Et_2O 5:1) afforded the desired product as a yellow oil (159 mg, 98%).

R_f = 0.36 (pentane/Et_2O 5:1); ^{1}H NMR (400 MHz, $CDCl_3$) δ = 8.17−8.12 (2H, m, H−C5), 7.43−7.37 (2H, m, H−C4), 4.65 (2H, dt, $^2J_{HF}$ = 46.7, $^3J_{HH}$ = 6.3, H−C1), 4.35 (2H, dt,

$^3J_{HF}$ = 19.2, $^3J_{HH}$ = 6.4, H−C2); ^{13}C NMR (101 MHz, $CDCl_3$) δ = 145.8 (C3), 145.7 (C6), 127.1 (2C, C4), 124.2 (2C, C5), 81.4 (d, $^1J_{CF}$ = 173.5, C1), 32.2 (d, $^2J_{CF}$ = 22.2, C2); ^{19}F NMR (282 MHz, $CDCl_3$) δ = −213.2 (1F, tt, $^2J_{HF}$ = 46.8, $^3J_{HF}$ = 19.2, F−C1); IR (ATR) $\tilde{\nu}$ = 3098w, 2958w, 2920w, 2850w, 1595m, 1578m, 1508m, 1479m, 1386m, 1365w, 1336m, 1289m, 1185m, 1113m, 1090m, 1060m, 1006m, 954m, 854m, 839m, 742m, 683m cm^{-1}; HRMS (ESI): *m/z*: 224.0160 ([M+Na]$^+$, calcd. for $C_8H_8FNO_2SNa^+$: 224.0152).

1-((2-Fluoroethyl)sulfinyl)-4-nitrobenzene (6)[2]

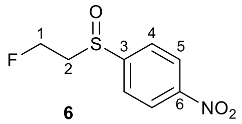

6

To a solution of **5** (20 mg, 0.1 mmol, 1.0 equiv.) in CH_2Cl_2 (0.5 ml) was added *m*CPBA (≤ 77%, 23 mg, 0.1 mmol, 1.0 equiv.) at 0 °C. The mixture was stirred under argon atmosphere for 19 h while warming to rt. The solvent was removed under reduced pressure to give a slightly yellow solid. Purification via column chromatography (silica, Cy/EtOAc 1:1 → 0:1) afforded the desired product as a white solid (37 mg, 68%).

R_f = 0.55 (EtOAc); m.p. = 67−68 °C; ^{1}H NMR (400 MHz, $CDCl_3$) δ = 8.42−8.38 (2H, m, H−C4/5), 7.87−7.83 (2H, m, H−C4/5), 4.90 (1H, dddd, $^2J_{HF}$ = 46.8, $^2J_{HH}$ = 10.7, $^3J_{HH}$ = 5.1, 4.2, H-C1), 4.87 (1H, dddd, $^2J_{HF}$ = 46.2, $^2J_{HH}$ = 10.7, $^3J_{HH}$ = 8.3, 3.2, H-C1), 3.28 (1H, dddd, $^2J_{HF}$ = 20.5, $^2J_{HH}$ = 14.1, $^3J_{HH}$ = 8.3, 4.1, H−C2), 3.09 (1H, dddd, $^2J_{HF}$ = 30.0, $^2J_{HH}$ = 14.1, $^3J_{HH}$ = 5.2, 3.2, H−C2); ^{13}C NMR (101 MHz, $CDCl_3$) δ = 151.1 (C3), 149.8 (C6), 125.1 (C4/5), 124.6 (C4/5), 75.9 (d, $^1J_{CF}$ = 171.5, C1), 57.8 (d, $^2J_{CF}$ = 19.9, C2); ^{19}F NMR (282 MHz, $CDCl_3$) δ = −210.16 (1F, tdd, $^2J_{HF}$ = 46.5, $^3J_{HF}$ = 30.0, 20.5, F−C1); IR (ATR) $\tilde{\nu}$ = 3101w, 3060w, 3028w, 3001w, 2966w, 2921w, 1946w, 1816w, 1698w, 1603m, 1586w, 1515m, 1479m, 1471m, 1427w, 1396m, 1347s, 1289m, 1227w, 1190w, 1176w, 1105m, 1082m, 1052m, 1032m, 1015m, 994s, 970m, 956m, 860m, 852s, 828m, 741s, 722s, 683m cm^{-1}; HRMS (ESI): *m/z*: 240.0110 ([M+Na]$^+$, calcd. for $C_8H_8FNO_3SNa^+$: 240.0101).

1-((2-Fluoroethyl)sulfonyl)-4-nitrobenzene (7)[2]

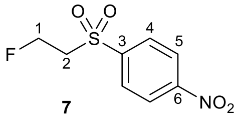

7

To a solution of **5** (40 mg, 0.2 mmol, 1.0 equiv.) in CH_2Cl_2 (1 ml) was added *m*CPBA (≤ 77%, 137 mg, 0.6 mmol, 3.0 equiv.) at 0 °C. The mixture was stirred under argon atmosphere for 20.5 h while warming to rt. The mixture was washed with aq. sat. $NaHCO_3$ (2 x 1 ml), brine, dried over Na_2SO_4, and concentrated under reduced pressure. Purification via column chromatography (silica, Cy/EtOAc 4:1) afforded the desired product as a white solid (42 mg, 90%).

R_f = 0.45 (Cy/EtOAc 4:1); m.p. = 111−113 °C; ^{1}H NMR (400 MHz, $CDCl_3$) δ = 8.44−8.40 (2H, m, H−C5), 8.18−8.12 (2H, m, H−C4), 4.87 (2H, dt, $^2J_{HF}$ = 46.7, $^3J_{HH}$ = 5.2, H−C1), 3.57 (2H, dt, $^3J_{HF}$ = 23.8, $^3J_{HH}$ = 5.2, H−C2); ^{13}C NMR (101 MHz, $CDCl_3$) δ = 151.2 (C3), 145.3 (C6), 129.9 (2C, C4), 124.6 (2C, C5), 77.2 (d, $^1J_{CF}$ = 173.0, C1), 57.1 (d, $^2J_{CF}$ = 21.4, C2); ^{19}F NMR (282 MHz, $CDCl_3$) δ = −220.10 (1F, tt, $^2J_{HF}$ = 46.7, $^3J_{HF}$ = 23.8, F−C1); IR (ATR) $\tilde{\nu}$ = 3104w, 3073w, 3039w, 2983w, 2929w, 1945w, 1693w, 1608w, 1575w, 1531m, 1478w, 1469w, 1417w, 1401w, 1380w, 1358m, 1349m, 1318m, 1307m, 1290m, 1240m, 1190m, 1178w, 1142m, 1110m, 1085m, 1063m, 1011m, 960m, 898w, 856m, 837w, 829w, 808w, 757m, 735m, 702m, 677m cm^{-1}; HRMS (ESI): *m/z*: 256.0067 ([M+Na]$^+$, calcd. for $C_8H_8FNO_4SNa^+$: 256.0050).

(3a'*R*,4*S*,7'*S*,7a'*S*)-2,2,2‘,2‘-Tetramethyltetrahydrospiro[[1,3]dioxolane-4,6‘-[1,3]dioxolo[4,5-*c*]pyran]-7'-ol (S5)[5]

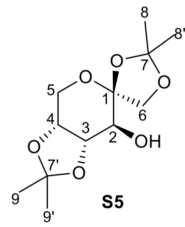

S5

To a suspension of D-fructose (18.016 g, 100.0 mmol, 1.0 equiv.) and 2,2-dimethoxypropane (7.4 ml, 60.0 mmol, 0.6 equiv.) in acetone (350 ml) was added perchloric acid (60%, 4.7 ml) dropwise at 0 °C under an atmosphere of argon. After stirring for 6 h at 0 °C, the mixture was quenched with aq. NH_4OH (30%, 10 ml), adjusting the pH to ≈ 9, and stirred for additional 10 min. The solvent was removed under reduced pressure and the obtained orange solid was dissolved in CH_2Cl_2 (100 ml) and H_2O (80 ml). The aqueous phase was extracted with CH_2Cl_2 (3 x 20 ml) and the combined organic layers were washed with brine, dried over Na_2SO_4, and concentrated under reduced pressure. The resulting residue was recrystallised from boiling hexanes/CH_2Cl_2 (v/v 4:1) to yield white needles (12.0 g, 46%).

m.p. = 116.3−118.5 °C; $[\alpha]_D^{23}$: −140.1° (*c* = 1.0, $CHCl_3$; [lit. $[\alpha]_D^{25}$: −144.2°, *c* = 1.0, $CHCl_3$]);); ^{1}H NMR (400 MHz, $CDCl_3$) δ = 4.20 (1H, ddd, *J* = 6.4, 2.6, 0.9, H−C4), 4.17 (1H, d, *J* = 8.8, H−C6), 4.11 (2H, m, H−C2/5), 3.98 (2H, m, H−C5/6), 3.65 (1H, d, *J* = 6.9, H−C3), 2.10 (1H, br, H−O), 1.52 (3H, s, H−8/8'), 1.50 (3H, s, H−C9/9'), 1.43 (3H, s, H−C9/9'), 1.36 (3H, s, H−C8/8'); ^{13}C−NMR (101 MHz, $CDCl_3$) δ = 112.0 (C7'), 109.5 (C7), 104.6 (C1), 77.3 (C2), 73.5 (C4), 72.4 (C6), 70.5 (C3), 60.9 (C5), 28.1 (C8/8'), 26.6 (C9/9'), 26.4 (C9/9'), 26.1 (C8/8'); IR (ATR) $\tilde{\nu}$ = 3456m, 2985w, 2944w, 2902w, 1485w, 1446w, 1387m, 1377m, 1367m, 1328w, 1274w, 1246m, 1217m, 1194m, 1181m, 1167m, 1113m, 1066m, 1043m, 1017m, 994m, 973m, 935m, 909m, 883m, 849m, 834m, 805m, 796m, 772m, 726m,

655w cm^{-1}; HRMS (ESI): *m/z*: 283.1160 ([M+Na]$^+$, calcd. for $C_{12}H_{20}O_6Na^+$: 283.1152). Analytical data in agreement with the literature.[5]

(3a'*R*,4*S*,7a'*R*)-2,2,2',2'-tetramethyldihydrospiro[[1,3]dioxolane-4,6'-[1,3]dioxolo[4,5-*c*]pyran]-7'(4'H)-one (S6)[5]

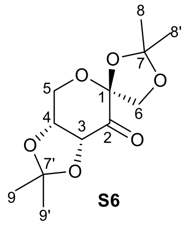

S6

A flask was charged with **S5** (6.00 g, 23.1 mmol, 1.0 equiv.) and powdered 3 Å molecular sieves (30 g, activated at 150 °C under hv over night) and CH_2Cl_2 (120 ml) was added under an atmosphere of argon. The suspension was stirred for 90 min, prior to the portionwise addition of PCC (13.42 g, 62.2 mmol, 2.7 equiv.) over 20 min. The mixture was stirred for 4 h while turning brown. After full consumption of starting material as indicated by TLC analysis, the mixture was filtered over celite and washed with copious amounts of Et_2O and the dark brown solution was concentrated under reduced pressure. The residue was filtered over silica (Cy/EtOAc 3:2) to yield the product as white solid (5.217 g, 88%) after concentration *in vacuo.*

R_f = 0.8 (Cy/EtOAc 3:2); m.p. = 98−99 °C; $[\alpha]_D^{25}$: −119.4° (*c* = 1.0, $CHCl_3$; [lit. $[\alpha]_D^{25}$: −125.4°, *c* = 1.0, $CHCl_3$]); ^{1}H NMR (400 MHz, $CDCl_3$) δ = 4.72 (1H, d, *J* = 5.5, H−C3), 4.60 (1H, d, *J* = 9.5, H−C6), 4.54 (1H, ddd, *J* = 5.6, 2.2, 1.0, H−C4), 4.38 (1H, dd, *J* = 13.5, 2.2, H−C5), 4.11 (1H, d, *J* = 13.5, H−C5), 3.98 (1H, d, *J* = 9.5, H−C6), 1.54 (3H, s, H−C8/8'), 1.45 (3H, s, H−C9/9'), 1.39 (6H, s, H−C8/8'/9'/9'); ^{13}C NMR (101 MHz, $CDCl_3$) δ = 197.1 (C2), 114.0 (C7), 110.8 (C7'), 104.2 (C1), 78.1 (C4), 76.0 (C3), 70.1 (C6), 60.3 (C5), 27.3 (C9/9'), 26.6 (C8/8'), 26.2 (C8/8'/9/9'), 26.1 (C8/8'/9/9'); IR (ATR) $\tilde{\nu}$ = 2988w, 2942w, 2891w, 1751m, 1463w, 1446w, 1382m, 1372m, 1341w, 1311w, 1284w, 1254m, 1226m, 1206m, 1182m, 1162m, 1136m, 1104m, 1093m, 1076m, 1050m, 1016m, 988m, 960m 034m, 923m, 893m, 870s, 855m 833m, 794m, 783m, 751m, 661w cm^{-1}; HRMS (ESI): *m/z*: 281.0992 ([M+Na]$^+$, calcd. for $C_{12}H_{18}O_6Na^+$: 281.0996). Analytical data in agreement with the literature.[5]

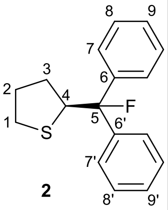

2

NMR Spectra of Key Compounds

^{1}H NMR (400 MHz, CD_2Cl_2)

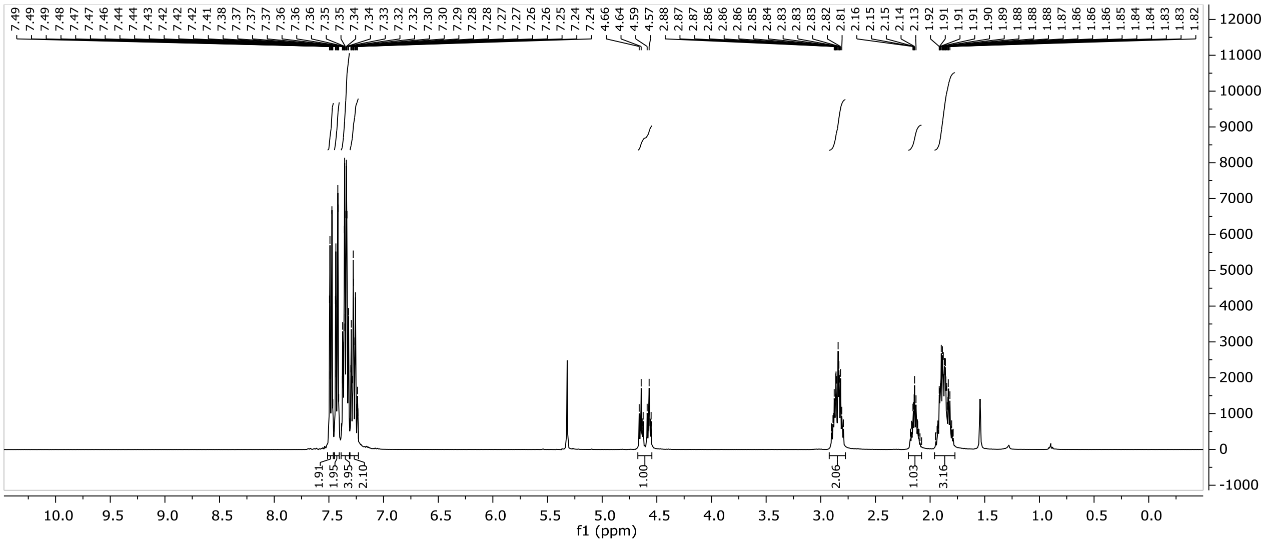

^{13}C NMR (101 MHz, CD_2Cl_2)

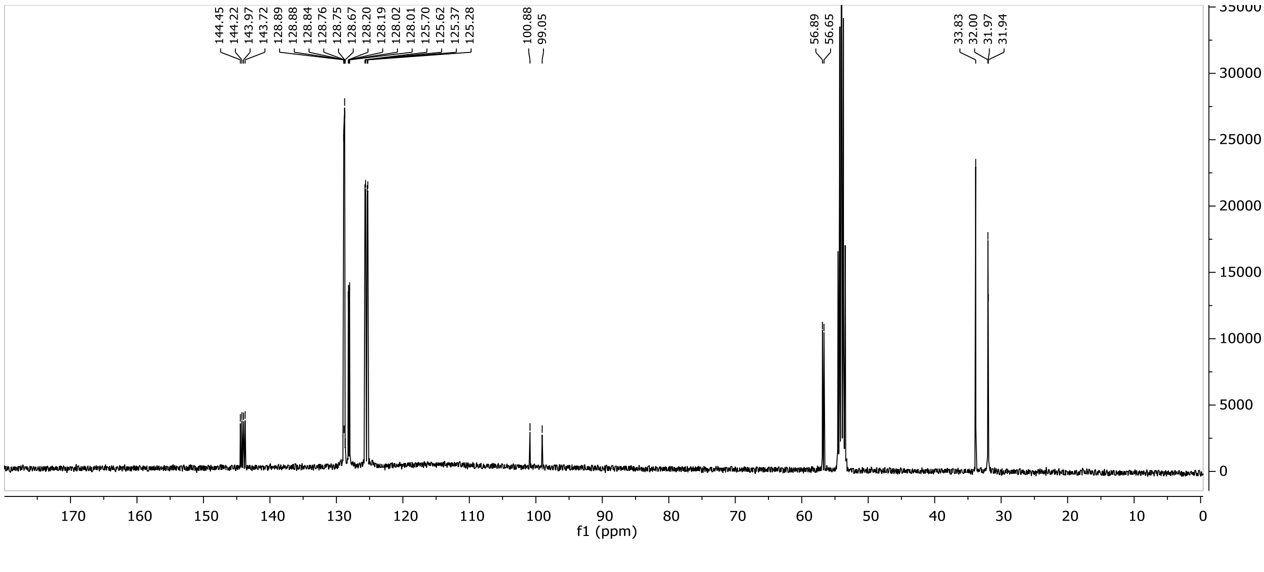

^{19}F NMR (282 MHz, CD_2Cl_2)

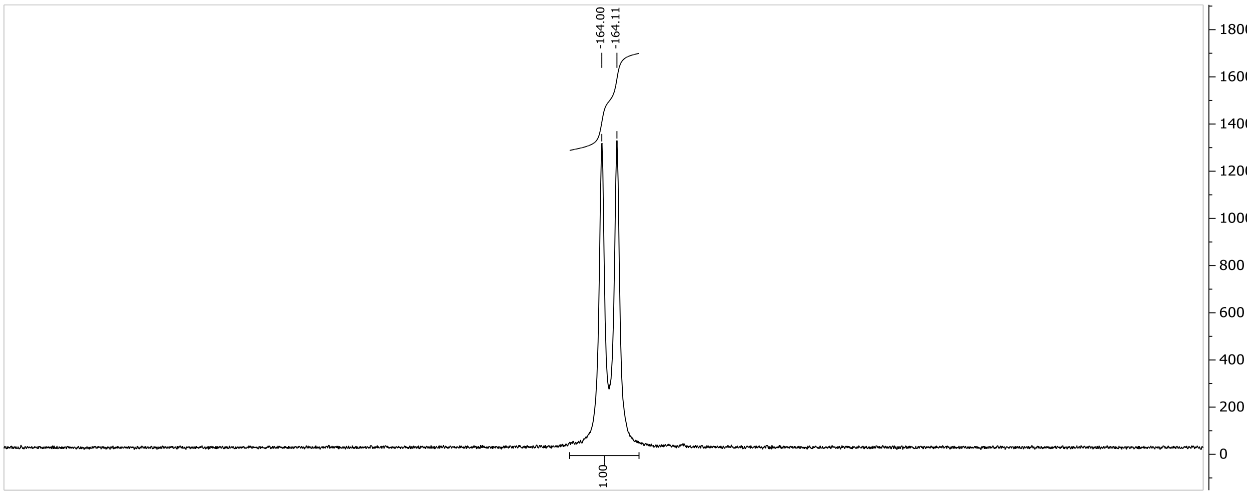

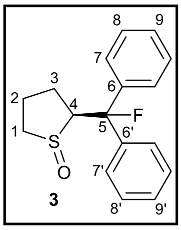

^{1}H NMR (400 MHz, CD_2Cl_2)

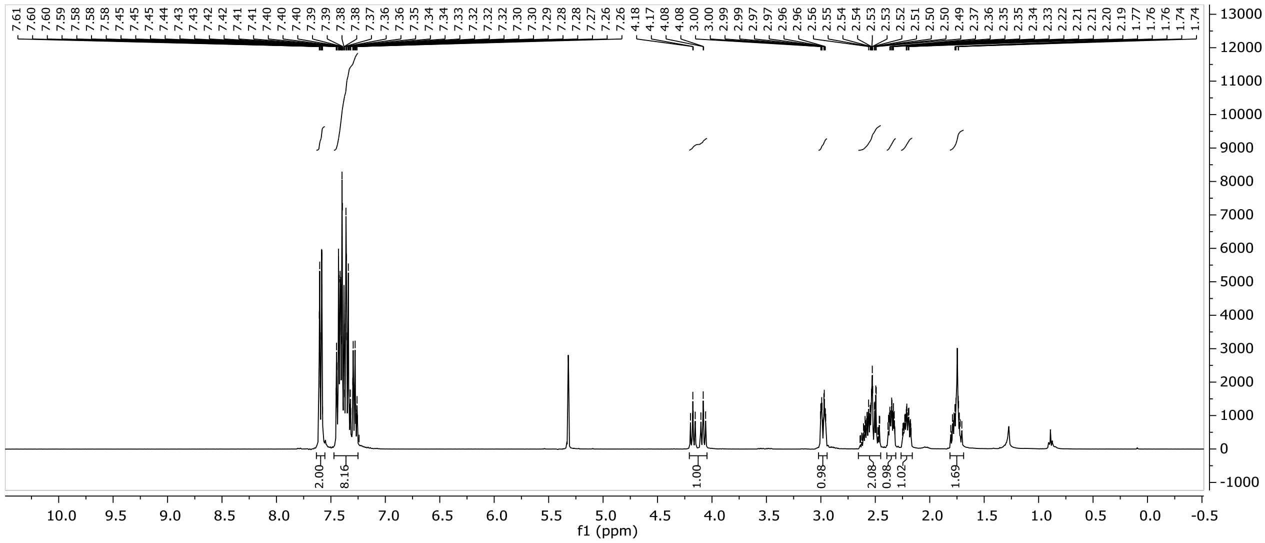

^{13}C NMR (101 MHz, CD_2Cl_2)

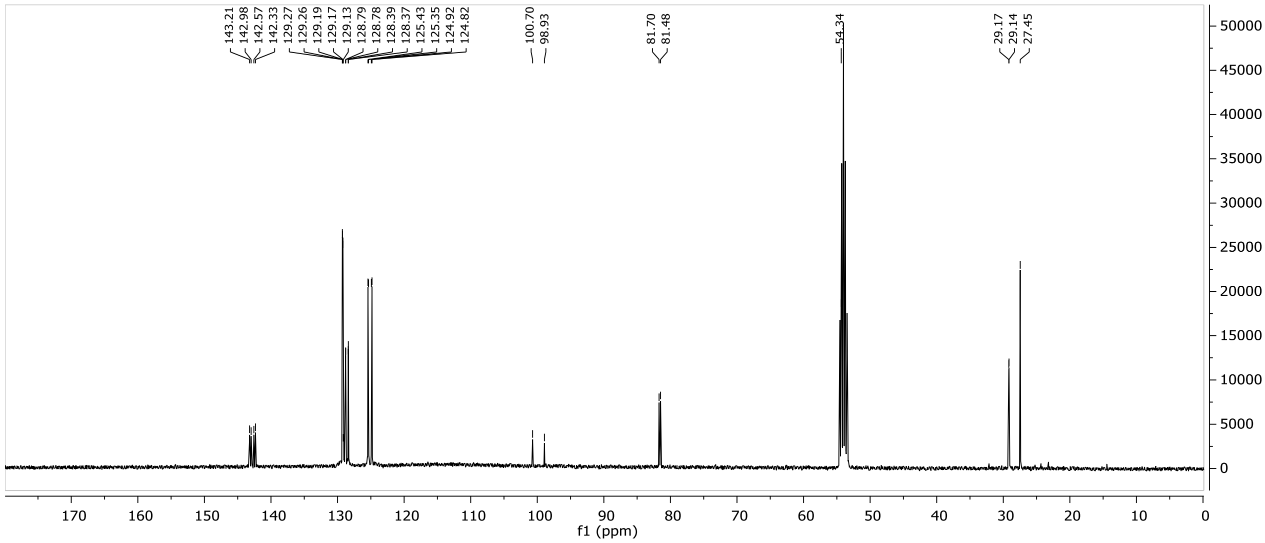

^{19}F NMR (282 MHz, CD_2Cl_2)

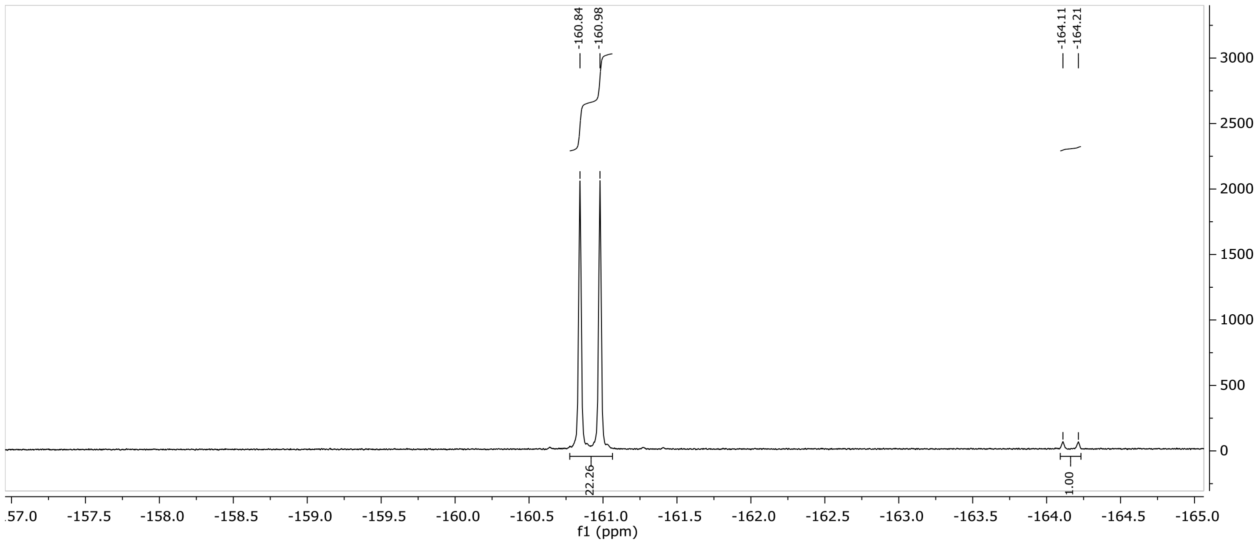

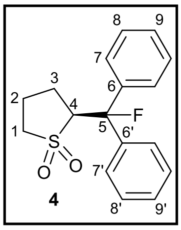

^{1}H NMR (600 MHz, CD_2Cl_2)

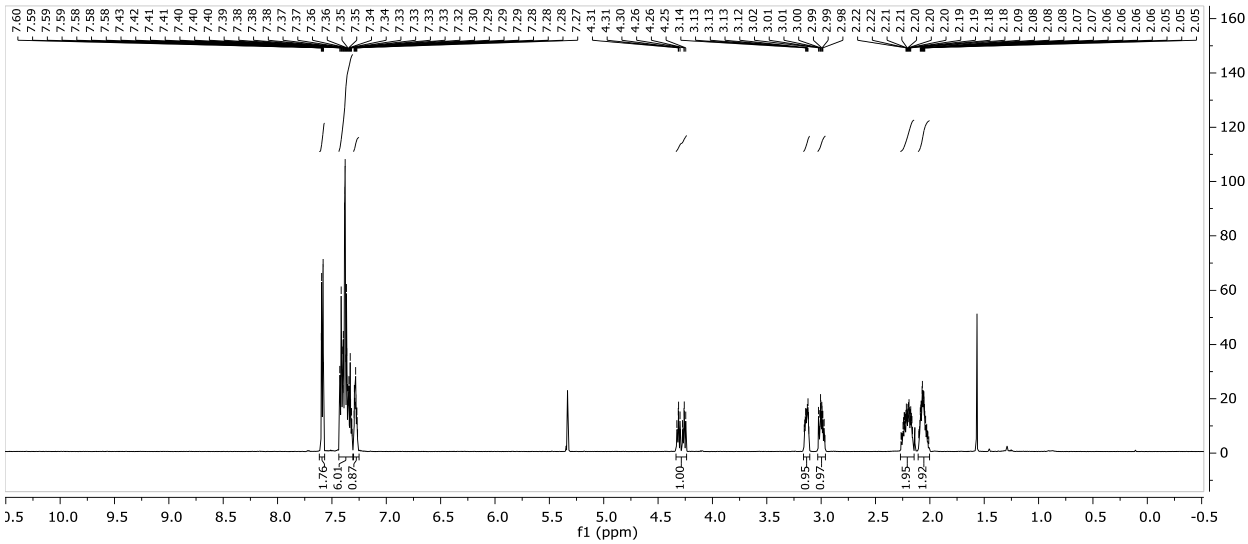

^{13}C NMR (151 MHz, CD_2Cl_2)

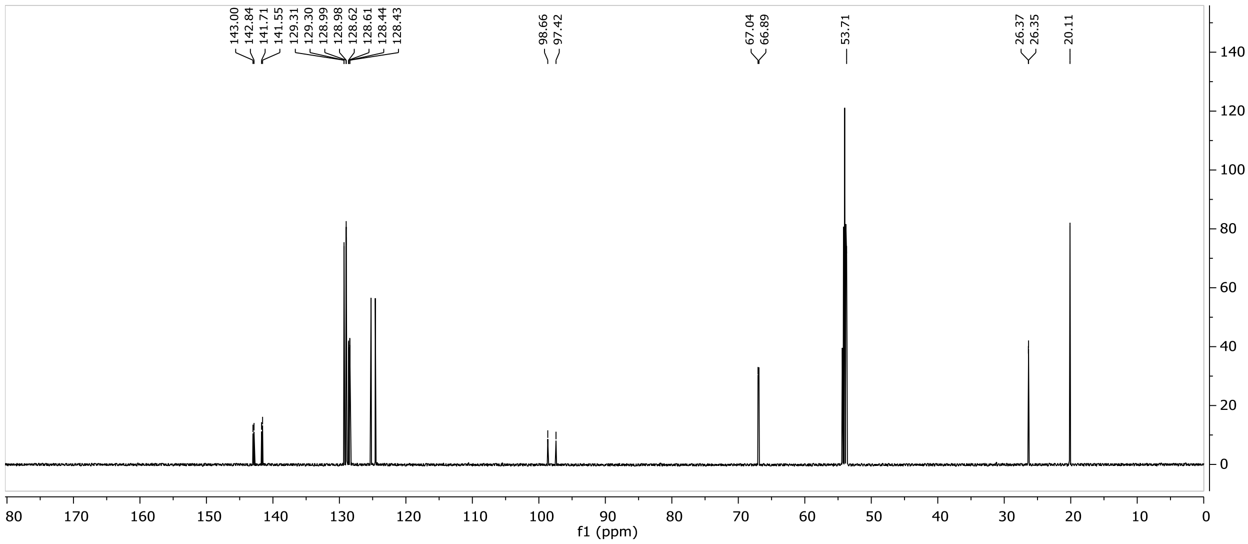

^{19}F NMR (282 MHz, CD_2Cl_2)

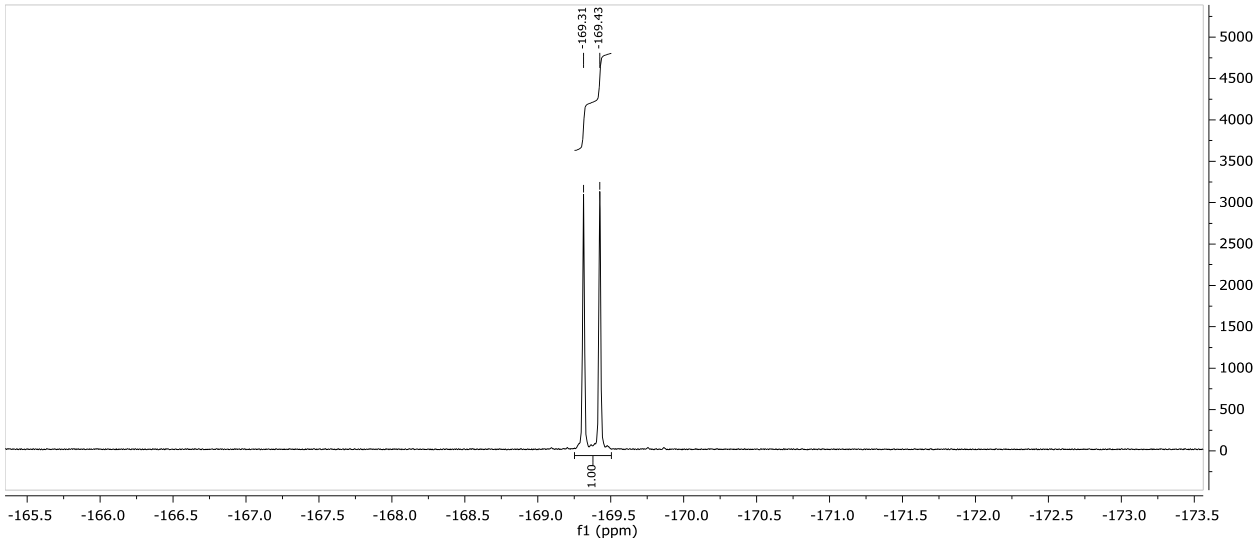

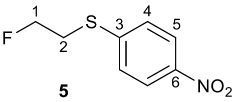

5

^{1}H NMR (400 MHz, $CDCl_3$)

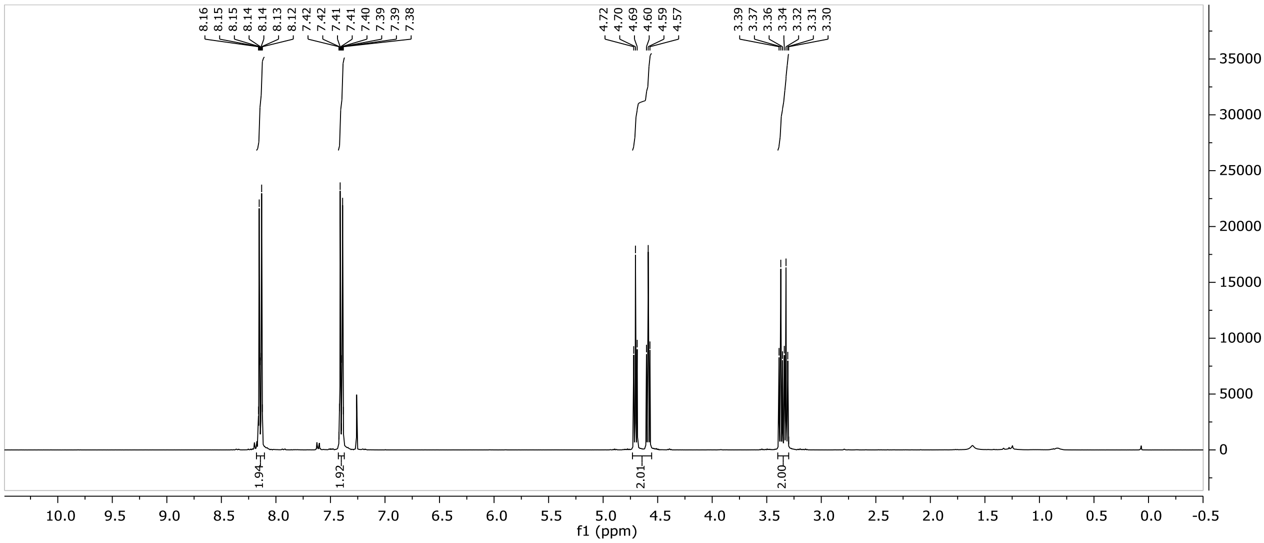

^{13}C NMR (101 MHz, $CDCl_3$)

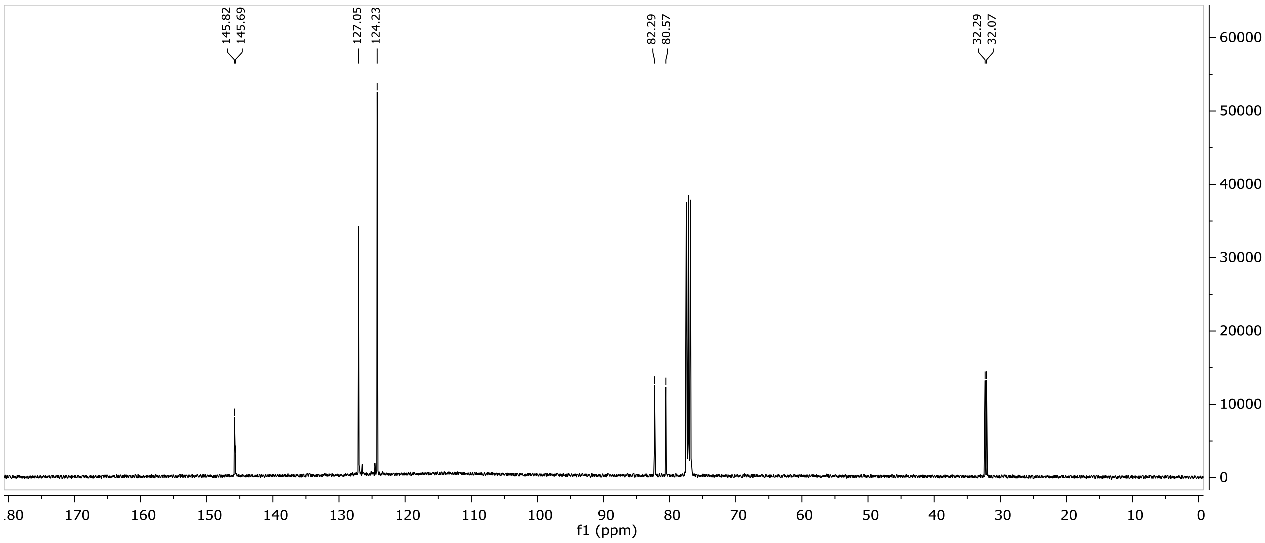

^{19}F NMR (282 MHz, $CDCl_3$)

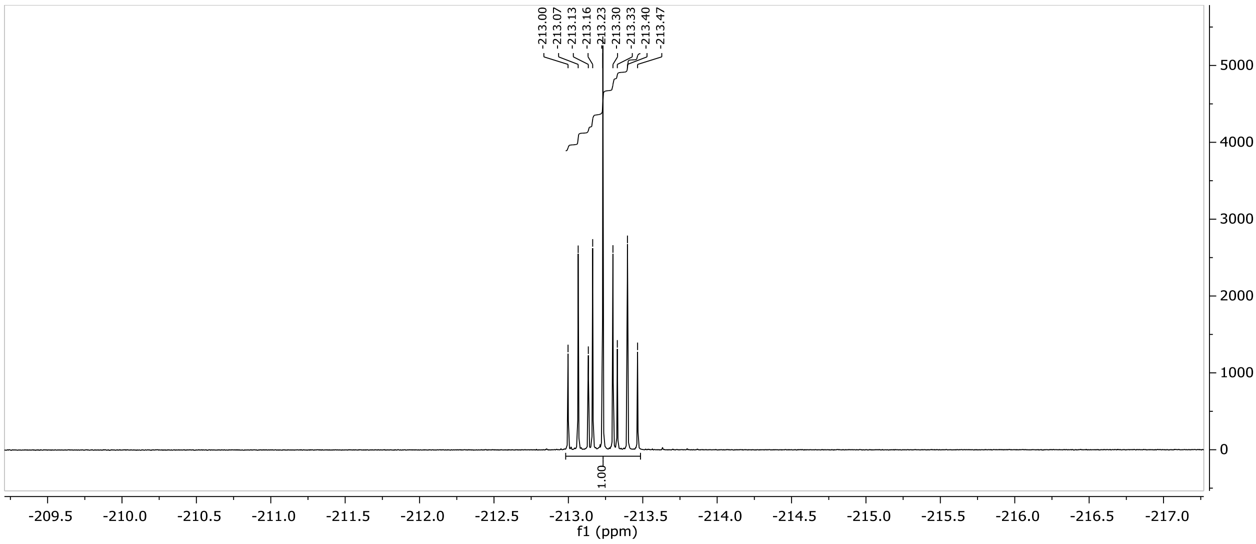

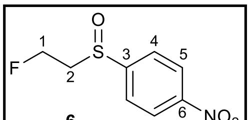

6

^{1}H NMR (400 MHz, $CDCl_3$)

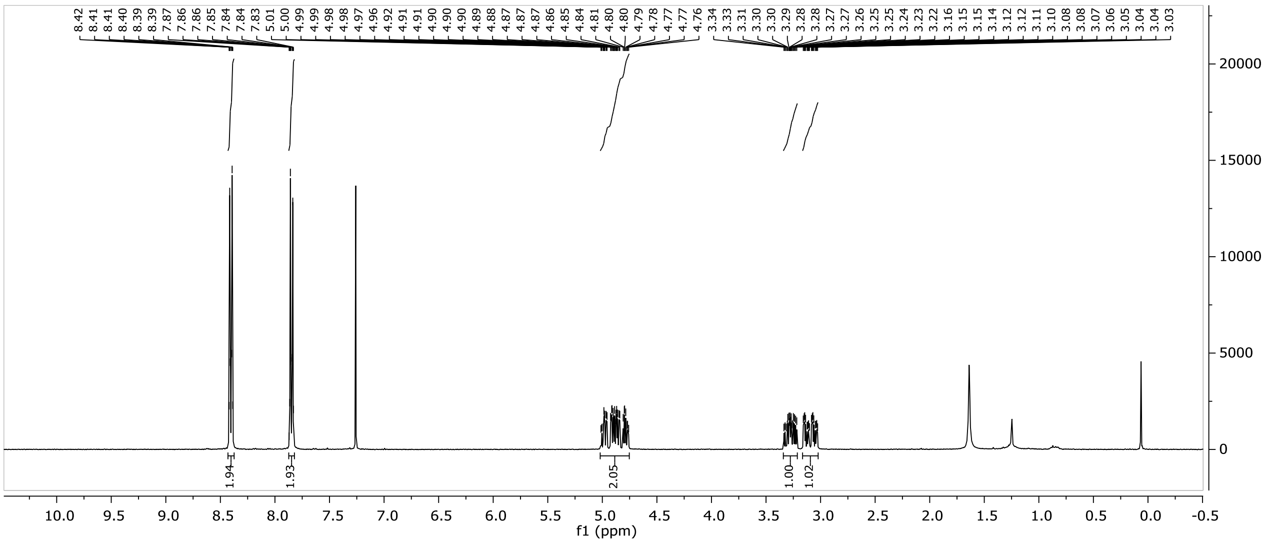

^{13}C NMR (101 MHz, $CDCl_3$)

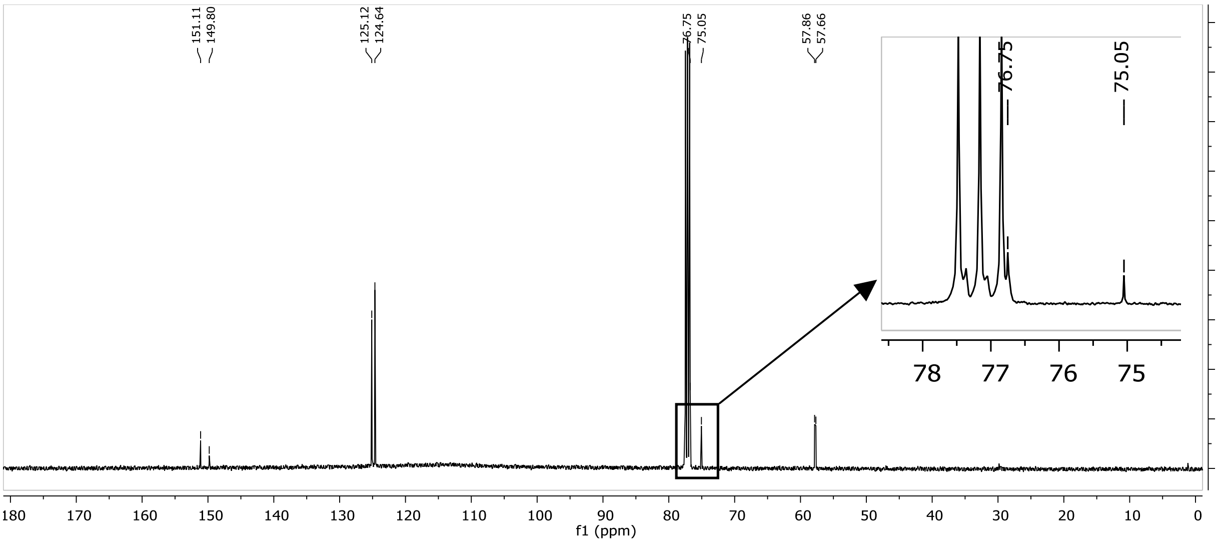

^{19}F NMR (282 MHz, $CDCl_3$)

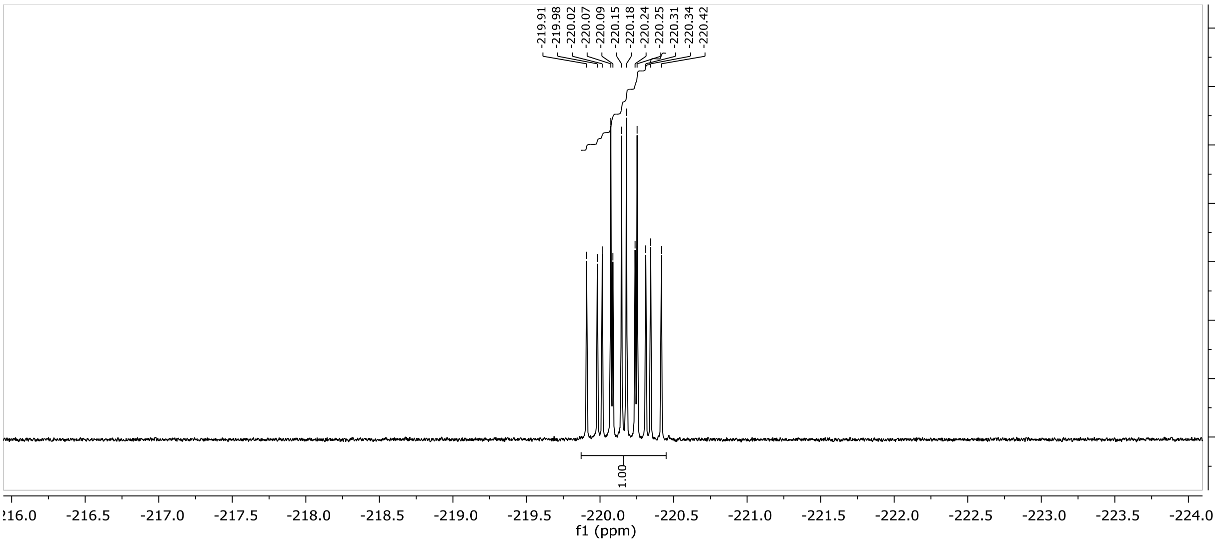

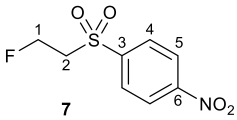

7

^{1}H NMR (400 MHz, $CDCl_3$)

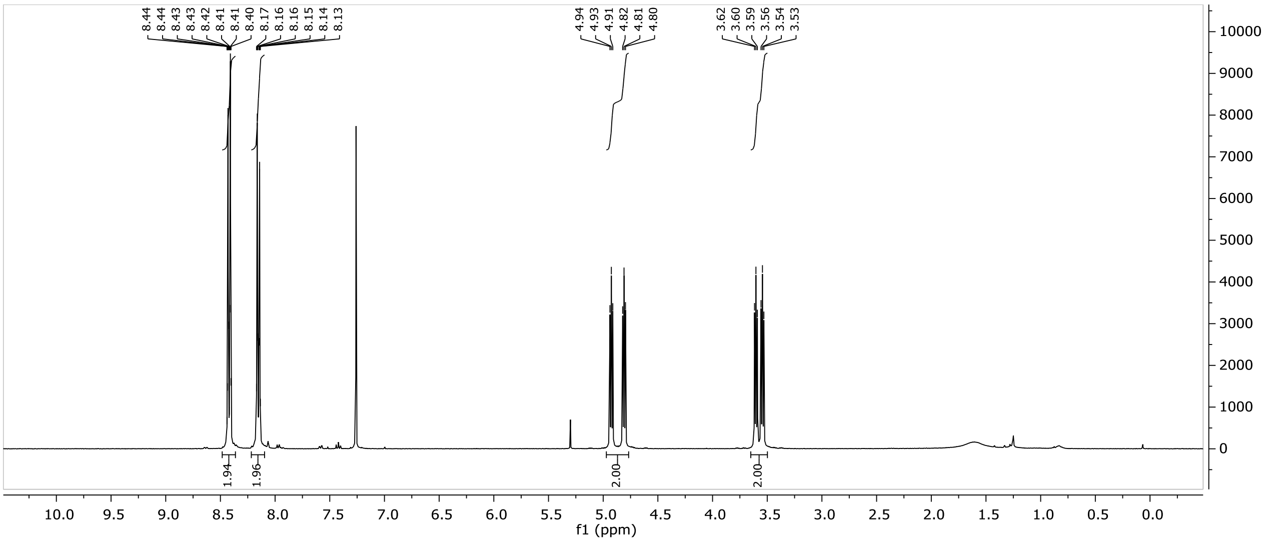

^{13}C NMR (101 MHz, $CDCl_3$)

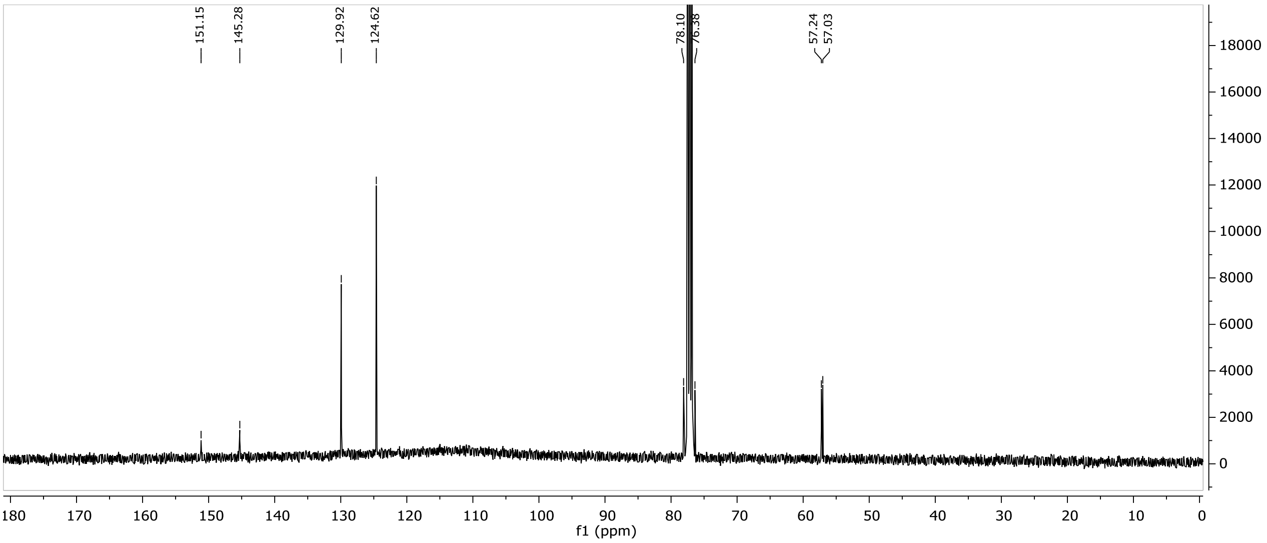

^{19}F NMR (282 MHz, $CDCl_3$)

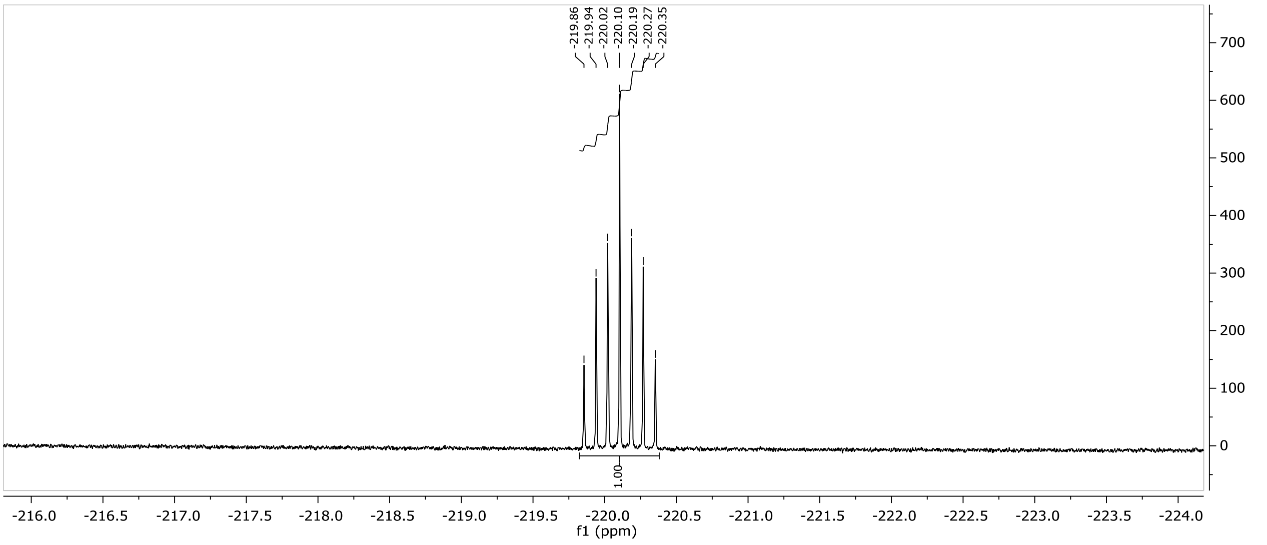

X-Ray Data of Key Compounds

X-Ray diffraction: Data sets for the compounds **3**, **4** and **7** were collected with a D8 Venture Dual Source 100 CMOS diffractometer. Programs used: data collection: APEX2 V2014.5-0 (Bruker AXS Inc., 2014); cell refinement: SAINT V8.34A (Bruker AXS Inc., 2013); data reduction: SAINT V8.34A (Bruker AXS Inc., 2013); absorption correction, SADABS V2014/2 (Bruker AXS Inc., 2014); structure solution SHELXT-2014 (Sheldrick, 2014); structure refinement SHELXL-2014 (Sheldrick, 2014). For the compounds **2** and **6** the data sets were collected with a Nonius KappaCCD diffractometer. Programs used: data collection, COLLECT (R. W. W. Hooft, Bruker AXS, 2008, Delft, The Netherlands); data reduction Denzo-SMN (Z. Otwinowski, W. Minor, *Methods Enzymol.* **1997**, *276*, 307-326); absorption correction, Denzo (Z. Otwinowski, D. Borek, W. Majewski, W. Minor, *Acta Crystallogr*. **2003**, *A59*, 228-234); structure solution SHELXS-97 (G. M. Sheldrick, *Acta Crystallogr*. **1990**, *A46*, 467-473); structure refinement SHELXL-97 (G. M. Sheldrick, *Acta Crystallogr*. **2008**, *A64*, 112-122). *R*-values are given for observed reflections, and wR^2 values are given for all reflections.

Exceptions and special features: For compound **6** one disordered over two positions NO_2 group and one $S{=}O(CH_2)(CH_2F)$ group were found in the asymmetrical unit. Several restraints (SADI, SIMU, ISOR and SAME) were used in order to improve refinement stability. The structure was refined as a 2-component inversion twin, whereby the BASF factor was refined to 0.14.

X-ray crystal structure analysis of 2 (gil7589): formula $C_{17}H_{17}FS$, *M* = 272.37, colourless crystal, 0.35 x 0.33 x 0.14 mm, *a* = 7.9685(7), *b* = 11.0136(9), *c* = 16.1416(14) Å, *V* = 1416.6(2) Å^3, ρ_{calc} = 1.277 gcm^{-3}, μ = 1.979 mm^{-1}, empirical absorption correction (0.544 ≤ T ≤ 0.769), *Z* = 4, orthorhombic, space group $P2_12_12_1$ (No. 19), λ = 1.54178 Å, *T* = 223(2) K, ω and φ scans, 7627 reflections collected (±*h*, ±*k*, ±*l*), 2377 independent (R_{int} = 0.029) and 2361 observed reflections [$I>2\sigma(I)$], 172 refined parameters, *R* = 0.028, wR^2 = 0.071, max. (min.) residual electron density 0.13 (-0.18) e.Å^{-3}, hydrogen atoms were calculated and refined as riding atoms. Flack parameter: 0.001(2).

X-ray crystal structure analysis of 3 (gil7668): formula $C_{17}H_{17}FOS$, *M* = 288.36, colourless crystal, 0.35 x 0.32 x 0.14 mm, *a* = 9.4841(3), *b* = 9.9993(3), *c* = 14.7157(5) Å, *V* = 1395.6(1) Å^3, ρ_{calc} = 1.372 gcm^{-3}, μ = 2.096 mm^{-1}, empirical absorption correction (0.601 ≤ T ≤ 0.753), *Z* = 4, orthorhombic, space group $P2_12_12_1$ (No. 19), λ = 1.54178 Å, *T* = 100(2) K, ω and φ scans, 6088 reflections collected (±*h*, ±*k*, ±*l*), 2488 independent (R_{int} = 0.025) and 2423 observed reflections [$I>2\sigma(I)$], 181 refined parameters, *R* = 0.026, wR^2 = 0.067, max.

(min.) residual electron density 0.23 (-0.26) e.Å^{-3}, hydrogen atoms were calculated and refined as riding atoms. Flack parameter: 0.03(1).

X-ray crystal structure analysis of 4 (gil7666): formula $C_{17}H_{17}FO_2S$, M = 304.36, colourless crystal, 0.34 x 0.21 x 0.10 mm, a = 14.6630(3), b = 9.5972(3), c = 10.7876(4) Å, β = 109.646(2) °, V = 1429.7(1) Å^3, ρ_{calc} = 1.414 gcm^{-3}, μ = 2.131 mm^{-1}, empirical absorption correction (0.849 ≤ T ≤ 1.000), Z = 4, monoclinic, space group $P2_1/c$ (No. 14), λ = 1.54178 Å, T = 100(2) K, ω and φ scans, 10126 reflections collected (±h, ±k, ±l), 2456 independent (R_{int} = 0.047) and 1909 observed reflections [I>2σ(I)], 190 refined parameters, R = 0.038, wR^2 = 0.092, max. (min.) residual electron density 0.24 (-0.37) e.Å^{-3}, hydrogen atoms were calculated and refined as riding atoms.

X-ray crystal structure analysis of 6 (gil7899): formula $C_8H_8FNO_3S$, M = 217.21, colourless crystal, 0.50 x 0.20 x 0.02 mm, a = 20.3421(7), b = 4.6595(2), c = 9.8133(4) Å, V = 930.1(1) Å^3, ρ_{calc} = 1.551 gcm^{-3}, μ = 0.343 mm^{-1}, empirical absorption correction (0.847 ≤ T ≤ 0.993), Z = 4, orthorhombic, space group Pna2$_1$ (No. 33), λ = 0.71073 Å, T = 223 (2) K, ω and φ scans, 7714 reflections collected (±h, ±k, ±l), 1589 independent (R_{int} = 0.063) and 1195 observed reflections [I>2σ(I)], 193 refined parameters, R = 0.059, wR^2 = 0.153, max. (min.) residual electron density 0.22 (-0.15) e.Å^{-3}, hydrogen atoms were calculated and refined as riding atoms.

X-ray crystal structure analysis of 7 (gil7727): formula $C_8H_8FNO_4S$, M = 233.21, colourless crystal, 0.31 x 0.21 x 0.08 mm, a = 7.2161(3), b = 7.3574(3), c = 9.1736(4) Å, α = 86.045(1), β = 76.993(1), γ = 78.266(1) °, V = 464.5(1) Å^3, ρ_{calc} = 1.667 gcm^{-3}, μ = 3.264 mm^{-1}, empirical absorption correction (0.430 ≤ T ≤ 0.767), Z = 2, triclinic, space group $P\bar{1}$ (No. 2), λ = 1.54178 Å, T = 100(2) K, ω and φ scans, 11607 reflections collected (±h, ±k, ±l), 1699 independent (R_{int} = 0.047) and 1631 observed reflections [I>2σ(I)], 136 refined parameters, R = 0.029, wR^2 = 0.075, max. (min.) residual electron density 0.36 (-0.32) e.Å^{-3}, hydrogen atoms were calculated and refined as riding atoms.

Computational Data

Coordinates and energies of conformers in Table 3:

8 (-*gauche*)

C	0.26479	0.70258	0.52639
H	1.35190	0.72192	0.43080
H	−0.03884	1.21160	1.44392
C	−0.26479	−0.70258	0.52639
H	−1.35190	−0.72192	0.43080
H	0.03884	−1.21160	1.44392
F	0.26479	−1.42771	−0.55923
F	−0.26479	1.42771	−0.55923

SCF energy: -278.391365 hartree
zero-point correction: +0.060621 hartree
enthalpy correction: +0.065911 hartree
free energy correction: +0.03425 hartree
quasiharmonic free energy correction: +0.03425 hartree

8 (*anti*)

C	−0.72320	0.23076	0.00000
H	−1.24953	−0.10435	0.89474
H	−1.24953	−0.10436	−0.89474
C	0.72320	−0.23076	0.00000
H	1.24953	0.10435	0.89474
H	1.24953	0.10436	−0.89474
F	0.72320	−1.63818	−0.00000
F	−0.72320	1.63818	−0.00000

SCF energy: -278.388106 hartree
zero-point correction: +0.060714 hartree
enthalpy correction: +0.066172 hartree
free energy correction: +0.034071 hartree
quasiharmonic free energy correction: +0.034071 hartree

9 (*anti,-gauche*)

C	1.71044	0.47080	-0.34816
H	1.75079	0.15079	-1.39107
H	2.36801	1.32986	-0.20079
C	0.30456	0.80390	0.09526
H	0.29294	1.03893	1.16212
H	-0.02168	1.68782	-0.46014
S	-0.86500	-0.57243	-0.24572
C	-2.42527	0.23935	0.24662
H	-2.61998	1.11739	-0.37073
H	-3.22055	-0.48985	0.09178
H	-2.40042	0.51988	1.30042
F	2.23916	-0.58669	0.42641

SCF energy: -616.661254 hartree
zero-point correction: +0.096872 hartree
enthalpy correction: +0.104628 hartree
free energy correction: +0.065343 hartree
quasiharmonic free energy correction: +0.066093 hartree

9 (*anti,anti*)

C	1.61090	-0.43192	0.00001
H	1.67913	-1.05531	-0.89291
C	0.36414	0.43119	-0.00000
H	0.34928	1.06310	0.89040
H	0.34929	1.06309	-0.89041
S	-1.12698	-0.64358	0.00000
C	-2.41766	0.64870	-0.00000
H	-2.34454	1.26816	-0.89438
H	-3.37652	0.13059	-0.00002
H	-2.34456	1.26814	0.89439
F	2.74402	0.41410	-0.00001
H	1.67913	-1.05529	0.89293

SCF energy: -616.660802 hartree
zero-point correction: +0.097059 hartree
enthalpy correction: +0.104914 hartree
free energy correction: +0.065358 hartree
quasiharmonic free energy correction: +0.065975 hartree

9 (*-gauche,-gauche*)

C	1.45884	0.59805	0.30302
H	1.05240	1.52053	-0.11494
H	2.22251	0.84202	1.04595
C	0.39448	-0.28720	0.91560
H	0.83498	-1.24259	1.20794
H	0.01192	0.19152	1.82156
S	-1.01610	-0.70532	-0.17946
C	-1.93422	0.87548	-0.17974
H	-1.35356	1.67813	-0.63498
H	-2.83053	0.71040	-0.77800
H	-2.22831	1.15204	0.83378
F	2.11485	-0.07611	-0.74924

SCF energy: -616.662051 hartree
zero-point correction: +0.096996 hartree
enthalpy correction: +0.104633 hartree
free energy correction: +0.065853 hartree
quasiharmonic free energy correction: +0.066297 hartree

9 (*+gauche,anti*)

C	-1.36142	0.19563	-0.46121
H	-1.47490	-0.36321	-1.39078
C	-0.42211	-0.50375	0.50222
H	-0.38606	0.01829	1.46056
H	-0.75972	-1.52600	0.68113
S	1.27632	-0.64795	-0.17264
C	1.92431	1.03741	0.12387
H	1.87753	1.28311	1.18536
H	2.96761	1.02232	-0.19224
H	1.39067	1.78605	-0.46076
F	-2.64809	0.28374	0.12993
H	-1.04822	1.21734	-0.67952

SCF energy: -616.662591 hartree
zero-point correction: +0.096974 hartree
enthalpy correction: +0.104770 hartree
free energy correction: +0.065603 hartree
quasiharmonic free energy correction: +0.065785 hartree

9 (+*gauche*,-*gauche*)

C	-1.65893	-0.34319	-0.15870
H	-1.69809	-0.62697	-1.21144
H	-2.57291	-0.66368	0.34832
C	-0.43928	-0.90902	0.53484
H	-0.38616	-0.56207	1.56956
H	-0.54068	-1.99739	0.55719
S	1.16647	-0.59190	-0.29205
C	1.51788	1.13182	0.21218
H	1.53381	1.21483	1.29974
H	2.50919	1.36651	-0.17662
H	0.78779	1.82113	-0.20665
F	-1.64606	1.07116	-0.11524

SCF energy: -616.662354 hartree
zero-point correction: +0.097014 hartree
enthalpy correction: +0.104648 hartree
free energy correction: +0.065989 hartree
quasiharmonic free energy correction: +0.066299 hartree

10 (*anti*,+*gauche*)

C	1.86238	-0.68779	-0.18929
H	2.55164	-1.40591	0.25950
C	0.47422	-0.87646	0.37968
H	0.16409	-1.90985	0.20468
H	0.43750	-0.66022	1.44985
C	-2.25820	-0.71233	0.15260
H	-2.22770	-0.79370	1.23968
H	-3.12669	-0.13432	-0.16072
H	-2.27415	-1.69542	-0.31968
S	-0.79080	0.20771	-0.43130
O	-0.79964	1.54328	0.31165
F	2.35274	0.59852	0.10576
H	1.88024	-0.79733	-1.27556

SCF energy: -691.867957 hartree
zero-point correction: +0.100457 hartree
enthalpy correction: +0.108984 hartree
free energy correction: +0.068108 hartree
quasiharmonic free energy correction: +0.068708 hartree

10 (*anti,anti*)

C	1.75444	0.33492	−0.21602
H	1.69162	1.28001	0.32317
C	0.58117	−0.57360	0.09787
H	0.65859	−1.51409	−0.45207
H	0.51155	−0.76758	1.17011
C	−2.11184	−1.08067	0.19942
H	−1.94356	−1.21650	1.26796
H	−3.12762	−0.73406	0.01316
H	−1.92500	−2.00170	−0.35416
F	2.93665	−0.30913	0.20827
H	1.85751	0.52163	−1.28631
S	−0.99914	0.22389	−0.42797
O	−1.18867	1.44355	0.47545

SCF energy: -691.868742 hartree
zero-point correction: +0.100418 hartree
enthalpy correction: +0.109150 hartree
free energy correction: +0.067447 hartree
quasiharmonic free energy correction: +0.068209 hartree

10 (*anti,-gauche*)

C	1.82478	−0.07345	0.57046
H	1.72988	0.94608	0.94734
C	0.49135	−0.78015	0.51136
H	0.57602	−1.74507	0.00687
H	0.10744	−0.92223	1.52476
C	−2.19341	−0.84884	−0.06164
H	−2.32533	−0.93969	1.01697
H	−3.05970	−0.36665	−0.51285
H	−2.02724	−1.82224	−0.52531
S	−0.75860	0.22537	−0.40992
O	−0.94922	1.50354	0.41095
F	2.38550	0.00706	−0.72230
H	2.52451	−0.63334	1.19316

SCF energy: -691.870677 hartree
zero-point correction: +0.100439 hartree
enthalpy correction: +0.109000 hartree
free energy correction: +0.067968 hartree
quasiharmonic free energy correction: +0.068567 hartree

10 (+*gauche*,+*gauche*)

C	-1.83130	0.24665	0.49545
H	-2.57744	-0.00264	1.25227
C	-0.54423	-0.49432	0.78097
H	-0.16871	-0.24239	1.77595
H	-0.70137	-1.57290	0.72615
C	1.23042	1.52964	0.06754
H	1.43266	1.56912	1.13848
H	2.11929	1.80655	-0.49830
H	0.40566	2.18776	-0.20841
S	0.83331	-0.18580	-0.41647
O	1.98818	-1.05408	0.09095
F	-2.35216	-0.15132	-0.75272
H	-1.70836	1.33003	0.46059

SCF energy: -691.868506 hartree
zero-point correction: +0.100439 hartree
enthalpy correction: +0.109040 hartree
free energy correction: +0.067651 hartree
quasiharmonic free energy correction: +0.068569 hartree

10 (+*gauche*,*anti*)

C	-1.71891	0.33782	-0.29110
H	-1.74486	0.28138	-1.38075
C	-0.60514	-0.50831	0.29779
H	-0.51695	-0.38693	1.37903
H	-0.77378	-1.56100	0.06389
C	1.32898	1.49202	0.28303
H	1.20106	1.44639	1.36498
H	2.35170	1.77004	0.03090
H	0.63520	2.20148	-0.17040
F	-2.94954	-0.17055	0.17688
H	-1.67105	1.38021	0.02460
S	1.05757	-0.16470	-0.43505
O	2.01422	-1.11134	0.28979

SCF energy: -691.866991 hartree
zero-point correction: +0.100500 hartree
enthalpy correction: +0.109209 hartree
free energy correction: +0.067629 hartree
quasiharmonic free energy correction: +0.068278 hartree

10 (+*gauche*,-*gauche*)

C	-1.83766	-0.67171	-0.15092
H	-1.84013	-0.89693	-1.21895
C	-0.48190	-0.89311	0.48134
H	-0.44998	-0.56532	1.52239
H	-0.24377	-1.95848	0.43809
C	0.76418	1.58891	0.21924
H	0.85619	1.56221	1.30562
H	1.57537	2.16870	-0.21949
H	-0.20238	1.98930	-0.08507
S	0.92596	-0.10799	-0.42975
O	2.19497	-0.68961	0.19965
F	-2.23874	0.67580	-0.01271
H	-2.58943	-1.28147	0.35263

SCF energy: -691.869502 hartree
zero-point correction: +0.100541 hartree
enthalpy correction: +0.109063 hartree
free energy correction: +0.068213 hartree
quasiharmonic free energy correction: +0.068848 hartree

10 (-*gauche*,*anti*)

C	1.49171	0.17139	-0.45543
H	1.39730	1.17459	-0.87032
C	0.64137	-0.03701	0.78166
H	0.93103	-0.96459	1.27927
H	0.72384	0.78543	1.49533
C	-1.52750	1.36653	-0.31551
H	-0.98382	1.47900	-1.25266
H	-2.60030	1.37632	-0.50399
H	-1.26803	2.15302	0.39406
F	2.84813	0.01141	-0.09083
H	1.27219	-0.57415	-1.21971
S	-1.16021	-0.26191	0.43437
O	-1.27194	-1.29340	-0.68983

SCF energy: -691.867574 hartree
zero-point correction: +0.100580 hartree
enthalpy correction: +0.109286 hartree
free energy correction: +0.067745 hartree
quasiharmonic free energy correction: +0.068417 hartree

11 (*anti,-gauche*)

C	1.93929	-0.65644	0.19934
H	1.82488	-0.61871	1.28306
C	0.62420	-0.93110	-0.49754
H	0.69874	-0.80817	-1.57859
H	0.30266	-1.94871	-0.26462
C	-2.21923	-0.69976	-0.42245
H	-2.27311	-1.64797	0.10943
H	-3.04863	-0.05152	-0.14055
H	-2.19739	-0.84411	-1.50156
F	2.46834	0.57776	-0.21396
H	2.66040	-1.42924	-0.07171
S	-0.72265	0.16828	0.07800
O	-0.66293	1.44409	-0.65665
O	-0.67279	0.20340	1.55241

SCF energy: -767.103631 hartree
zero-point correction: +0.105893 hartree
enthalpy correction: +0.115073 hartree
free energy correction: +0.072565 hartree
quasiharmonic free energy correction: +0.073186 hartree

11 (*anti,anti*)

C	-1.88341	-0.36242	-0.00001
H	-1.86857	-0.98523	0.89389
C	-0.75746	0.66120	-0.00003
H	-0.79384	1.28387	-0.89480
H	-0.79386	1.28391	0.89472
C	2.04321	1.20154	-0.00004
H	1.89657	1.79387	0.90151
H	3.03016	0.73906	-0.00001
H	1.89659	1.79381	-0.90163
S	0.86732	-0.16099	0.00001
O	1.00583	-0.90524	-1.26542
O	1.00582	-0.90515	1.26549
F	-3.09809	0.34811	0.00001
H	-1.86860	-0.98523	-0.89390

SCF energy: -767.103036 hartree
zero-point correction: +0.106243 hartree
enthalpy correction: +0.115521 hartree
free energy correction: +0.072337 hartree
quasiharmonic free energy correction: +0.073327 hartree

11 (+*gauche*,-*gauche*)

C	1.92515	-0.63582	0.09034
H	1.81193	-0.85807	1.15150
C	0.64318	-0.86595	-0.67646
H	0.70798	-0.52112	-1.70954
H	0.39769	-1.93081	-0.67614
C	-0.68969	1.67195	-0.24591
H	-0.68371	1.83900	-1.32164
H	-1.56901	2.12669	0.20993
H	0.22550	2.03019	0.22052
F	2.32036	0.71377	-0.01847
H	2.72435	-1.24362	-0.33634
S	-0.83891	-0.09533	0.06206
O	-0.79112	-0.32050	1.51946
O	-2.00229	-0.59976	-0.69107

SCF energy: -767.106048 hartree
zero-point correction: +0.106198 hartree
enthalpy correction: +0.115237 hartree
free energy correction: +0.073265 hartree
quasiharmonic free energy correction: +0.073732 hartree

11 (-*gauche*,*anti*)

C	-1.83143	0.27752	0.32453
H	-1.74397	1.36361	0.28090
C	-0.78218	-0.43016	-0.51853
H	-0.90251	-1.51387	-0.46239
H	-0.82030	-0.12109	-1.56398
C	1.32597	1.53831	-0.40212
H	0.65808	2.22068	0.12077
H	2.35504	1.69280	-0.07803
H	1.24441	1.64156	-1.48271
S	0.92394	-0.15686	0.05355
O	1.79048	-1.05798	-0.72464
O	0.93386	-0.25932	1.52447
F	-3.09247	-0.05459	-0.20596
H	-1.81036	-0.05817	1.36031

SCF energy: -767.102789 hartree
zero-point correction: +0.106311 hartree
enthalpy correction: +0.115457 hartree
free energy correction: +0.073132 hartree
quasiharmonic free energy correction: +0.073314 hartree

11 (*-gauche,-gauche*)

C	-1.90507	0.64027	-0.22651
H	-1.68675	1.36509	0.55929
C	-0.67466	0.19562	-0.99252
H	-0.87310	-0.72154	-1.55054
H	-0.36174	0.96650	-1.69943
C	1.39974	1.39719	0.60854
H	0.64895	1.83610	1.26325
H	2.30667	1.17471	1.17050
H	1.62226	2.04186	-0.24009
F	-2.53286	-0.45896	0.37623
H	-2.61963	1.07839	-0.92578
S	0.80838	-0.19309	-0.00110
O	1.81014	-0.72333	-0.94439
O	0.42797	-1.01660	1.15904

SCF energy: -767.102338 hartree
zero-point correction: +0.106006 hartree
enthalpy correction: +0.115130 hartree
free energy correction: +0.072889 hartree
quasiharmonic free energy correction: +0.073318 hartree

Coordinates and energies of conformers in Table 4:

5 (*anti,+gauche*)

C	1.50465	-1.12100	-0.00123
C	0.13477	-0.90364	0.05036
C	-0.37348	0.40443	0.08621
C	0.52145	1.49221	0.06621
C	1.88667	1.28029	0.01462
C	2.37009	-0.03002	-0.01803
H	1.90131	-2.12625	-0.02878
H	-0.52396	-1.76008	0.05991
H	0.14062	2.50632	0.09172
H	2.57639	2.11235	-0.00009
N	3.80992	-0.25983	-0.07122
O	4.55581	0.71908	-0.08809
O	4.21590	-1.42159	-0.09656
S	-2.09158	0.81318	0.15137
C	-2.93300	-0.81912	0.19660
H	-2.71988	-1.37084	-0.72010
H	-2.57205	-1.38748	1.05660
C	-4.42696	-0.62984	0.35410
H	-4.68081	-0.01143	1.21672
H	-4.91351	-1.60277	0.44255
F	-4.96567	0.00314	-0.78420

SCF energy: -1013.012629 hartree
zero-point correction: +0.152206 hartree
enthalpy correction: +0.165424 hartree
free energy correction: +0.110805 hartree
quasiharmonic free energy correction: +0.113776 hartree

5 (*anti,anti*)

C	1.42806	-1.10113	-0.00043
C	0.07946	-0.77145	-0.00061
C	-0.31793	0.57382	-0.00033
C	0.66236	1.58468	0.00011
C	2.00674	1.26064	0.00029
C	2.38020	-0.08517	0.00003
H	1.74104	-2.13581	-0.00064
H	-0.64803	-1.57021	-0.00100
H	0.36605	2.62696	0.00035
H	2.76256	2.03327	0.00065
N	3.79809	-0.43389	0.00025
O	4.62223	0.47982	0.00059
O	4.10613	-1.62520	-0.00013
S	-1.99863	1.12555	-0.00056

C	-2.96968	-0.43235	-0.00022
H	-2.73249	-1.01074	-0.89394
H	-2.73149	-1.01089	0.89314
C	-4.44666	-0.07147	0.00063
H	-4.73578	0.48390	-0.89272
H	-4.73484	0.48346	0.89455
F	-5.18418	-1.27265	0.00071

SCF energy: -1013.011544 hartree
zero-point correction: +0.152135 hartree
enthalpy correction: +0.165523 hartree
free energy correction: +0.109657 hartree
quasiharmonic free energy correction: +0.113380 hartree

5 (+*gauche*,+*gauche*)

C	1.29526	-1.00252	-0.43986
C	-0.04818	-0.68457	-0.58137
C	-0.50831	0.60464	-0.27295
C	0.41034	1.57206	0.17784
C	1.75141	1.26277	0.31508
C	2.18484	-0.02830	0.00637
H	1.65371	-1.99458	-0.67620
H	-0.72106	-1.45083	-0.93851
H	0.06680	2.56995	0.42334
H	2.45810	2.00374	0.66126
N	3.59748	-0.36414	0.15502
O	4.36675	0.51330	0.54549
O	3.95692	-1.50926	-0.11725
S	-2.18425	1.15464	-0.43980
C	-3.15973	-0.35486	-0.79729
H	-4.10491	0.03427	-1.18082
H	-2.69649	-0.92146	-1.60676
C	-3.42403	-1.25306	0.39450
H	-2.51230	-1.56020	0.90792
H	-3.98193	-2.13721	0.07698
F	-4.22168	-0.57758	1.33828

SCF energy: -1013.012601 hartree
zero-point correction: +0.152254 hartree
enthalpy correction: +0.165346 hartree
free energy correction: +0.111309 hartree
quasiharmonic free energy correction: +0.113998 hartree

5 (+*gauche,anti*)

C	1.19466	-0.97450	-0.40347
C	-0.12397	-0.54706	-0.47502
C	-0.45763	0.77538	-0.14841
C	0.55787	1.66448	0.24957
C	1.87488	1.24528	0.31771
C	2.18312	-0.07625	-0.00929
H	1.45708	-1.99277	-0.65368
H	-0.87776	-1.25475	-0.79026
H	0.31117	2.68732	0.50840
H	2.65747	1.92503	0.62356
N	3.57057	-0.52715	0.06495
O	4.42821	0.28369	0.41249
O	3.81974	-1.69697	-0.22367
S	-2.09162	1.46240	-0.23570
C	-3.19988	0.04013	-0.54314
H	-4.12528	0.49971	-0.89403
H	-2.81076	-0.57887	-1.35167
C	-3.45964	-0.76911	0.71590
H	-3.94544	-0.16746	1.48459
H	-2.55219	-1.21957	1.11894
F	-4.33806	-1.82788	0.39300

SCF energy: -1013.012872 hartree
zero-point correction: +0.152440 hartree
enthalpy correction: +0.165546 hartree
free energy correction: +0.111689 hartree
quasiharmonic free energy correction: +0.114161 hartree

5 (+*gauche,-gauche*)

C	1.13450	0.96321	0.34926
C	-0.20387	0.59782	0.37128
C	-0.58117	-0.72008	0.07523
C	0.41103	-1.66415	-0.25267
C	1.74592	-1.30301	-0.28371
C	2.09927	0.01264	0.02293
H	1.43054	1.97827	0.57417
H	-0.94459	1.35040	0.59470
H	0.13176	-2.68623	-0.47960
H	2.50982	-2.02529	-0.53463
N	3.50551	0.40074	-0.00303
O	4.34063	-0.45226	-0.30339
O	3.79586	1.56367	0.27692
S	-2.24312	-1.33671	0.08105
C	-3.29633	0.00952	0.74973
H	-4.12941	-0.52151	1.21702
H	-2.76416	0.53569	1.54278

C	-3.86917	0.96254	-0.27776
H	-4.57873	1.64268	0.19956
H	-4.35646	0.43283	-1.09726
F	-2.85337	1.75924	-0.84514

SCF energy: -1013.012657 hartree
zero-point correction: +0.152247 hartree
enthalpy correction: +0.165329 hartree
free energy correction: +0.111482 hartree
quasiharmonic free energy correction: +0.113954 hartree

6 (*anti,+gauche*)

C	-1.84433	1.15268	0.30242
C	-0.47397	1.22302	0.07296
C	0.19285	0.10404	-0.41413
C	-0.48165	-1.08098	-0.71014
C	-1.84998	-1.15861	-0.48371
C	-2.50671	-0.03873	0.02271
H	-2.39148	2.00141	0.68773
H	0.07944	2.13446	0.26307
H	0.04206	-1.93622	-1.12079
H	-2.40251	-2.06183	-0.69982
N	-3.96009	-0.11709	0.26133
O	-4.52748	-1.17654	0.01605
O	-4.52733	0.87994	0.69455
S	1.98198	0.25441	-0.77067
O	2.34216	1.71110	-0.52552
C	2.56995	-0.72667	0.69562
H	2.36716	-0.12191	1.58191
H	1.96821	-1.63784	0.72663
C	4.02945	-1.10953	0.58575
H	4.25847	-1.59956	-0.36279
H	4.29759	-1.77264	1.41080
F	4.85307	0.02620	0.67836

SCF energy: -1088.211112 hartree
zero-point correction: +0.155546 hartree
enthalpy correction: +0.169793 hartree
free energy correction: +0.112621 hartree
quasiharmonic free energy correction: +0.116277 hartree

6 (*anti,anti*)

C	-1.85193	0.99057	0.64551
C	-0.50163	1.21149	0.39516
C	0.18074	0.34776	-0.45482
C	-0.45635	-0.71886	-1.08867
C	-1.80475	-0.94552	-0.84181
C	-2.47730	-0.08688	0.02512
H	-2.41178	1.63797	1.30550
H	0.02122	2.04596	0.84611
H	0.08032	-1.36553	-1.77311
H	-2.33004	-1.76399	-1.31289
N	-3.90985	-0.32387	0.28691
O	-4.44913	-1.26967	-0.27750
O	-4.48768	0.43559	1.05667
S	1.94023	0.69372	-0.81218
O	2.26821	2.03215	-0.16662
C	2.62647	-0.60983	0.31312
H	2.24907	-0.39991	1.31522
H	2.24453	-1.56883	-0.04190
C	4.14379	-0.57774	0.27268
H	4.54071	0.37234	0.63008
H	4.53446	-0.79634	-0.72250
F	4.62128	-1.58272	1.13800

SCF energy: -1088.211338 hartree
zero-point correction: +0.155523 hartree
enthalpy correction: +0.169918 hartree
free energy correction: +0.112468 hartree
quasiharmonic free energy correction: +0.11578 hartree

6 (*anti,-gauche*)

C	-1.89129	1.15334	0.26248
C	-0.51979	1.28180	0.06931
C	0.21035	0.18262	-0.37072
C	-0.39923	-1.04068	-0.65084
C	-1.76813	-1.17672	-0.45676
C	-2.48965	-0.07551	-0.00042
H	-2.48819	1.98541	0.60826
H	-0.01490	2.22281	0.25063
H	0.17566	-1.88020	-1.02380
H	-2.27176	-2.11098	-0.66001
N	-3.94373	-0.21578	0.20297
O	-4.45320	-1.30931	-0.01831
O	-4.56940	0.76722	0.58490
S	1.99982	0.39345	-0.67429
O	2.31700	1.86550	-0.44726
C	2.56414	-0.48906	0.86159

H	2.08801	0.01261	1.70678
H	2.21573	−1.52117	0.79457
C	4.06716	−0.41875	0.99656
H	4.37973	−0.87171	1.93904
H	4.43744	0.60586	0.93990
F	4.67675	−1.14631	−0.04550

SCF energy: -1088.213744 hartree
zero-point correction: +0.155586 hartree
enthalpy correction: +0.169810 hartree
free energy correction: +0.113156 hartree
quasiharmonic free energy correction: +0.116195 hartree

6 (+*gauche*,+*gauche*)

C	1.70422	−1.13676	0.49064
C	0.34492	−1.37007	0.32112
C	−0.42824	−0.43403	−0.36136
C	0.13337	0.71743	−0.91266
C	1.49271	0.95973	−0.74309
C	2.25181	0.02904	−0.03979
H	2.33161	−1.84020	1.01951
H	−0.11495	−2.27478	0.69968
H	−0.46987	1.41642	−1.47983
H	1.95902	1.84382	−1.15391
N	3.69500	0.28129	0.13929
O	4.16117	1.31386	−0.32962
O	4.35311	−0.55337	0.75026
S	−2.20422	−0.78140	−0.61897
O	−2.39301	−2.27465	−0.37962
C	−2.84961	0.00622	0.93885
H	−3.89154	−0.31774	0.96667
H	−2.30766	−0.45530	1.76700
C	−2.74974	1.51161	1.00560
H	−1.72769	1.87859	0.90316
H	−3.16412	1.86477	1.95178
F	−3.50878	2.09245	−0.02995

SCF energy: -1088.211407 hartree
zero-point correction: +0.155930 hartree
enthalpy correction: +0.170014 hartree
free energy correction: +0.114084 hartree
quasiharmonic free energy correction: +0.116601 hartree

6 (+*gauche,anti*)

C	-1.65652	-0.90677	-0.82862
C	-0.31459	-1.21663	-0.64222
C	0.39416	-0.58524	0.37631
C	-0.21486	0.32399	1.24021
C	-1.55652	0.64177	1.05716
C	-2.25076	0.02231	0.02191
H	-2.23552	-1.37325	-1.61302
H	0.17815	-1.95005	-1.26878
H	0.33928	0.77576	2.05488
H	-2.05889	1.34526	1.70558
N	-3.67601	0.35387	-0.17392
O	-4.18522	1.17259	0.58343
O	-4.27616	-0.20477	-1.08518
S	2.14638	-1.03437	0.64268
O	2.36134	-2.37316	-0.04800
C	2.90698	0.20540	-0.51136
H	3.95420	-0.10117	-0.54187
H	2.45009	0.04751	-1.48925
C	2.79351	1.64596	-0.04784
H	3.20774	1.79230	0.95095
H	1.77059	2.02041	-0.08594
F	3.55292	2.44156	-0.93038

SCF energy: -1088.209089 hartree
zero-point correction: +0.155791 hartree
enthalpy correction: +0.170109 hartree
free energy correction: +0.113109 hartree
quasiharmonic free energy correction: +0.116076 hartree

6 (*eclipsed,-gauche*)

C	1.78620	-1.23381	0.28123
C	0.42469	-1.48018	0.13784
C	-0.41067	-0.45352	-0.28954
C	0.08183	0.81276	-0.60631
C	1.44051	1.06498	-0.46418
C	2.26915	0.03593	-0.02059
H	2.46229	-2.00709	0.61752
H	0.00697	-2.45639	0.35068
H	-0.58125	1.59773	-0.94779
H	1.85544	2.03596	-0.69438
N	3.71159	0.30159	0.13036
O	4.12068	1.42769	-0.13265
O	4.42989	-0.61607	0.51274
S	-2.18330	-0.83648	-0.53378
O	-2.34316	-2.31800	-0.21500
C	-2.87860	0.09165	0.93622

H	-3.56764	-0.63456	1.37338
H	-2.08042	0.30837	1.64500
C	-3.63729	1.33172	0.52786
H	-4.10241	1.79322	1.40053
H	-4.39644	1.12619	-0.22842
F	-2.75100	2.28255	-0.02494

SCF energy: -1088.210837 hartree
zero-point correction: +0.155470 hartree
enthalpy correction: +0.169737 hartree
free energy correction: +0.112429 hartree
quasiharmonic free energy correction: +0.116090 hartree

7 (*anti,-gauche*)

C	2.03195	-1.21267	-0.07252
C	0.65497	-1.21540	-0.26543
C	-0.02261	0.00117	-0.31868
C	0.63678	1.22214	-0.19511
C	2.01358	1.22889	0.00148
C	2.68154	0.01037	0.06186
H	2.59251	-2.13548	-0.03005
H	0.12057	-2.14846	-0.38648
H	0.08856	2.15240	-0.26325
H	2.55953	2.15602	0.10121
N	4.14692	0.01529	0.27351
O	4.70007	1.09800	0.42051
O	4.72602	-1.06369	0.29217
S	-1.81757	-0.00194	-0.56204
O	-2.18334	1.28010	-1.18126
O	-2.17944	-1.26442	-1.22715
C	-2.47953	-0.06538	1.14543
H	-1.86926	-0.79788	1.67749
H	-2.32833	0.92322	1.57989
C	-3.93127	-0.49449	1.18606
H	-4.22944	-0.64866	2.22451
H	-4.10374	-1.40457	0.61086
F	-4.75519	0.50887	0.65237

SCF energy: -1163.442236 hartree
zero-point correction: +0.161027 hartree
enthalpy correction: +0.175906 hartree
free energy correction: +0.117704 hartree
quasiharmonic free energy correction: +0.121035 hartree

7 (*anti,anti*)

C	2.01005	−1.09352	−0.54465
C	0.64821	−0.96878	−0.79622
C	−0.01016	0.18943	−0.38804
C	0.65159	1.23096	0.25913
C	2.01361	1.10937	0.51186
C	2.66308	−0.05226	0.10692
H	2.55644	−1.97443	−0.84959
H	0.11247	−1.75348	−1.31388
H	0.11803	2.12669	0.54875
H	2.56274	1.89710	1.00728
N	4.11372	−0.18301	0.37504
O	4.67601	0.74453	0.94372
O	4.67143	−1.21203	0.01447
S	−1.78151	0.34873	−0.71635
O	−2.10935	1.78222	−0.75773
O	−2.10844	−0.50545	−1.86862
C	−2.56069	−0.37105	0.76482
H	−2.19984	−1.39770	0.83852
H	−2.20917	0.21349	1.61598
C	−4.07954	−0.32585	0.65449
H	−4.44622	−0.92280	−0.17993
H	−4.45323	0.69513	0.58275
F	−4.59858	−0.88518	1.83576

SCF energy: -1163.441253 hartree
zero-point correction: +0.161013 hartree
enthalpy correction: +0.176112 hartree
free energy correction: +0.116793 hartree
quasiharmonic free energy correction: +0.120700 hartree

7 (+*gauche*,-*gauche*)

C	−1.75336	−0.95115	−0.85932
C	−0.38178	−1.15757	−0.76824
C	0.34317	−0.46617	0.20052
C	−0.26348	0.41560	1.09083
C	−1.63652	0.61895	1.00807
C	−2.35209	−0.06539	0.03122
H	−2.34891	−1.46900	−1.59728
H	0.10821	−1.85685	−1.43286
H	0.32363	0.92675	1.84168
H	−2.14315	1.29234	1.68439
N	−3.81330	0.15188	−0.06072
O	−4.32862	0.92503	0.73723
O	−4.42834	−0.45256	−0.93069
S	2.12630	−0.75161	0.32742
O	2.56457	−0.25492	1.64167

O	2.37504	-2.15262	-0.04811
C	2.91626	0.27673	-0.96093
H	3.93275	-0.12396	-0.99769
H	2.41475	0.05274	-1.90330
C	2.95714	1.75762	-0.66194
H	3.60067	2.25732	-1.38789
H	3.31026	1.96031	0.34950
F	1.67074	2.31465	-0.78361

SCF energy: -1163.443703 hartree
zero-point correction: +0.161014 hartree
enthalpy correction: +0.175880 hartree
free energy correction: +0.117709 hartree
quasiharmonic free energy correction: +0.121080 hartree

7 (+*gauche,anti*)

C	-1.85971	-0.52489	-1.12048
C	-0.50644	-0.83446	-1.05642
C	0.19823	-0.54933	0.11255
C	-0.41440	0.02297	1.22490
C	-1.77068	0.32769	1.16659
C	-2.46344	0.05082	-0.00658
H	-2.43902	-0.73237	-2.00868
H	-0.01691	-1.30459	-1.89933
H	0.15339	0.21245	2.12620
H	-2.28148	0.76636	2.01174
N	-3.90766	0.37270	-0.07069
O	-4.42828	0.86572	0.92191
O	-4.50182	0.12862	-1.11322
S	1.95779	-0.97017	0.20184
O	2.35689	-0.91414	1.61642
O	2.16115	-2.20345	-0.57075
C	2.82402	0.36206	-0.68596
H	3.84234	-0.01955	-0.78844
H	2.36598	0.43273	-1.67351
C	2.80640	1.69386	0.04852
H	3.30085	1.62807	1.01681
H	1.79769	2.09121	0.16736
F	3.52430	2.61480	-0.73561

SCF energy: -1163.441135 hartree
zero-point correction: +0.161021 hartree
enthalpy correction: +0.176031 hartree
free energy correction: +0.117351 hartree
quasiharmonic free energy correction: +0.120681 hartree

7 (+*gauche*,+*gauche*)

C	1.88607	−1.03049	0.70292
C	0.52222	−1.27271	0.58815
C	−0.24407	−0.45171	−0.23818
C	0.31609	0.59728	−0.96379
C	1.68152	0.84071	−0.85386
C	2.43651	0.02415	−0.01924
H	2.51273	−1.64748	1.33075
H	0.06910	−2.09779	1.12233
H	−0.30027	1.20402	−1.61393
H	2.15218	1.64260	−1.40436
N	3.88967	0.28274	0.10072
O	4.35949	1.21512	−0.53914
O	4.54200	−0.44910	0.83467
S	−2.01972	−0.77556	−0.39256
O	−2.49206	−0.08663	−1.60107
O	−2.22612	−2.22586	−0.25364
C	−2.76819	−0.00698	1.08681
H	−3.82843	−0.25688	1.00515
H	−2.34625	−0.54575	1.93718
C	−2.55889	1.48354	1.25719
H	−1.51587	1.78197	1.13842
H	−2.90170	1.77412	2.25209
F	−3.31641	2.20488	0.32463

SCF energy: -1163.441359 hartree
zero-point correction: +0.160909 hartree
enthalpy correction: +0.175830 hartree
free energy correction: +0.117170 hartree
quasiharmonic free energy correction: +0.12096 hartree

Coordinates and energies of conformers in Table 5:

2 (+*gauche*)

S	-2.36097	-1.41185	0.98343
F	0.18329	-0.00010	2.09410
C	-2.99969	-2.22537	-0.54693
H	-3.76876	-2.94175	-0.25881
H	-3.44952	-1.47463	-1.20002
C	-1.78422	-2.88500	-1.20043
H	-1.98826	-3.13866	-2.24454
H	-1.54175	-3.81279	-0.67384
C	-0.61771	-1.89775	-1.08250
H	-0.75063	-1.07887	-1.79156
H	0.33529	-2.37400	-1.32123
C	-0.59610	-1.38272	0.37097
H	-0.06674	-2.11474	0.98309
C	0.14966	-0.05789	0.65391
C	-0.46419	1.27052	0.21627
C	-1.47037	1.40010	-0.74377
H	-1.90272	0.53138	-1.21602
C	-1.95012	2.66001	-1.10816
H	-2.73178	2.73587	-1.85562
C	-1.43918	3.80884	-0.51133
H	-1.81690	4.78559	-0.79171
C	-0.43789	3.69091	0.45341
H	-0.03169	4.57654	0.92897
C	0.04623	2.43553	0.80752
H	0.82583	2.35591	1.55518
C	1.60359	-0.20047	0.19545
C	2.02851	0.31006	-1.03526
H	1.33882	0.86649	-1.65823
C	3.33983	0.11687	-1.46800
H	3.65299	0.52031	-2.42433
C	4.24621	-0.58165	-0.67180
H	5.26779	-0.72534	-1.00496
C	3.83104	-1.08899	0.55896
H	4.52958	-1.62822	1.18892
C	2.51740	-0.90562	0.98756
H	2.20639	-1.29702	1.94785

SCF energy: -1156.309788 hartree
zero-point correction: +0.294743 hartree
enthalpy correction: +0.312037 hartree
free energy correction: +0.249520 hartree
quasiharmonic free energy correction: +0.252753 hartree

2 (*anti*)

S	0.95962	−1.87942	−1.19701
F	−0.25339	0.08720	2.16527
C	2.76016	−2.21047	−0.93508
H	3.02385	−3.10953	−1.49181
H	3.33945	−1.37018	−1.32113
C	2.92857	−2.36345	0.57573
H	3.97792	−2.25880	0.86583
H	2.59317	−3.35674	0.88921
C	2.05945	−1.27991	1.21906
H	2.50786	−0.30143	1.03150
H	1.97847	−1.40797	2.30014
C	0.66626	−1.35435	0.57085
H	0.09836	−2.15531	1.04659
C	−0.18581	−0.06665	0.72975
C	−1.64360	−0.20030	0.29060
C	−2.19311	−1.38981	−0.19520
H	−1.58013	−2.27163	−0.32897
C	−3.54751	−1.46501	−0.52825
H	−3.95165	−2.39516	−0.91135
C	−4.37386	−0.35726	−0.36533
H	−5.42569	−0.41772	−0.62086
C	−3.83773	0.83305	0.12979
H	−4.47211	1.70196	0.26371
C	−2.48615	0.91015	0.44831
H	−2.07964	1.84067	0.82639
C	0.47491	1.19647	0.18275
C	0.37248	1.51704	−1.17655
H	−0.20101	0.88235	−1.83922
C	0.99493	2.65431	−1.68690
H	0.90480	2.88530	−2.74241
C	1.72057	3.49675	−0.84485
H	2.19925	4.38515	−1.24124
C	1.81692	3.19203	0.51112
H	2.37012	3.84383	1.17809
C	1.20132	2.04834	1.02137
H	1.27688	1.82559	2.07731

SCF energy: -1156.312293 hartree
zero-point correction: +0.294681 hartree
enthalpy correction: +0.311956 hartree
free energy correction: +0.249701 hartree
quasiharmonic free energy correction: +0.252617 hartree

2 (-*gauche*)

S	2.78396	0.38218	-0.11750
F	0.31924	-0.21494	1.75527
C	3.82763	-1.12075	0.14610
H	4.79456	-0.95501	-0.32875
H	3.97938	-1.26837	1.21663
C	3.04488	-2.27830	-0.47409
H	3.41532	-3.24138	-0.11137
H	3.16213	-2.26475	-1.56217
C	1.57678	-2.06450	-0.09814
H	1.44319	-2.24822	0.97080
H	0.91495	-2.74359	-0.63995
C	1.22118	-0.60102	-0.41473
H	0.98446	-0.50891	-1.47556
C	0.01262	-0.05838	0.37924
C	-1.23417	-0.90634	0.09541
C	-1.84780	-0.85466	-1.16195
H	-1.46580	-0.19228	-1.92984
C	-2.96466	-1.63927	-1.43933
H	-3.42693	-1.58557	-2.41854
C	-3.49124	-2.48265	-0.46070
H	-4.36347	-3.08980	-0.67458
C	-2.89001	-2.53290	0.79473
H	-3.29262	-3.18102	1.56520
C	-1.76650	-1.75250	1.07203
H	-1.30705	-1.80053	2.04999
C	-0.27477	1.42763	0.15961
C	0.01271	2.07399	-1.04663
H	0.49692	1.54447	-1.85755
C	-0.31086	3.41967	-1.22620
H	-0.07586	3.90389	-2.16723
C	-0.92347	4.13786	-0.20254
H	-1.17125	5.18403	-0.34127
C	-1.21275	3.50015	1.00421
H	-1.68701	4.04948	1.80976
C	-0.89537	2.15624	1.18199
H	-1.12260	1.67159	2.12295

SCF energy: -1156.31508 hartree
zero-point correction: +0.294719 hartree
enthalpy correction: +0.312023 hartree
free energy correction: +0.249229 hartree
quasiharmonic free energy correction: +0.252758 hartree

3a (+*gauche*)

C	3.28779	−1.02254	0.86165
H	4.24595	−1.50785	0.67726
H	3.45916	0.01846	1.14828
F	−0.18062	−0.28595	−1.90807
O	2.64902	−2.31503	−1.45268
S	2.41214	−0.96450	−0.76626
C	2.36359	−1.73864	1.84065
H	2.69294	−1.59604	2.87258
H	2.36108	−2.81339	1.63956
C	0.97218	−1.13855	1.60623
H	0.95085	−0.11804	1.99385
H	0.19665	−1.70219	2.12838
C	0.72197	−1.14030	0.09173
H	0.45672	−2.14044	−0.25815
C	−0.30388	−0.13765	−0.48089
C	0.03606	1.31612	−0.15299
C	−0.38684	1.89096	1.05217
H	−1.00028	1.32117	1.73985
C	−0.04487	3.20317	1.37227
H	−0.38263	3.63117	2.30924
C	0.71626	3.96551	0.48677
H	0.97637	4.98894	0.73184
C	1.13080	3.40496	−0.71944
H	1.71335	3.99154	−1.42080
C	0.79632	2.08838	−1.03743
H	1.11413	1.66782	−1.98152
C	−1.76215	−0.49228	−0.18139
C	−2.75976	0.39965	−0.60166
H	−2.47991	1.33584	−1.06932
C	−4.10658	0.09673	−0.43162
H	−4.86133	0.79838	−0.76800
C	−4.48391	−1.10469	0.17021
H	−5.53294	−1.34044	0.30822
C	−3.50324	−1.99962	0.58769
H	−3.78327	−2.93859	1.05118
C	−2.15200	−1.69884	0.40644
H	−1.41797	−2.42472	0.72955

SCF energy: -1231.519248 hartree
zero-point correction: +0.298077 hartree
enthalpy correction: +0.316365 hartree
free energy correction: +0.252032 hartree
quasiharmonic free energy correction: +0.255031 hartree

3a (*anti*)

C	-0.96652	3.21430	-0.82049
H	-0.62420	4.11651	-1.32714
H	-1.80931	2.79037	-1.37215
F	-0.00887	-0.52521	2.22105
O	1.72611	2.75203	-0.76296
S	0.41497	1.98886	-0.96910
C	-1.27831	3.37076	0.66535
H	-2.23929	3.86924	0.81317
H	-0.51156	3.98321	1.14772
C	-1.27907	1.95119	1.24297
H	-2.17095	1.41429	0.91021
H	-1.28489	1.95416	2.33478
C	-0.01244	1.25437	0.72992
H	0.85894	1.60042	1.29101
C	0.00475	-0.29271	0.80333
C	1.33018	-0.87289	0.30256
C	1.42695	-1.63403	-0.86532
H	0.54254	-1.85590	-1.44728
C	2.66224	-2.12286	-1.29320
H	2.71757	-2.71062	-2.20241
C	3.81380	-1.86681	-0.55412
H	4.77212	-2.25076	-0.88484
C	3.72510	-1.11562	0.61838
H	4.61419	-0.91429	1.20499
C	2.49541	-0.61998	1.04079
H	2.44051	-0.04665	1.95789
C	-1.23517	-0.98319	0.24021
C	-1.89757	-1.95332	0.99966
H	-1.53799	-2.19619	1.99011
C	-3.02216	-2.60558	0.49506
H	-3.52224	-3.35187	1.10199
C	-3.50211	-2.30215	-0.77717
H	-4.37704	-2.80879	-1.16802
C	-2.84527	-1.34109	-1.54348
H	-3.20371	-1.09661	-2.53689
C	-1.72171	-0.68840	-1.03904
H	-1.22192	0.04054	-1.66559

SCF energy: -1231.518799 hartree
zero-point correction: +0.297808 hartree
enthalpy correction: +0.316256 hartree
free energy correction: +0.250211 hartree
quasiharmonic free energy correction: +0.254959 hartree

3a (-*gauche*)

C	3.21116	−1.83419	0.61054
H	4.29006	−1.86055	0.45837
H	2.99261	−1.91682	1.67866
F	0.06278	−0.32347	1.77191
O	3.33249	0.16073	−1.23348
S	2.64772	−0.15104	0.10411
C	2.40368	−2.82714	−0.21902
H	2.51723	−3.84479	0.16226
H	2.75486	−2.82114	−1.25446
C	0.94925	−2.35127	−0.13215
H	0.55032	−2.58153	0.85827
H	0.31415	−2.84832	−0.86772
C	0.93151	−0.82787	−0.35741
H	0.89212	−0.59740	−1.42236
C	−0.18444	−0.07697	0.39011
C	−1.55851	−0.66887	0.05801
C	−2.09103	−0.52037	−1.22816
H	−1.54727	0.03159	−1.98600
C	−3.32987	−1.06947	−1.54818
H	−3.72756	−0.94612	−2.54910
C	−4.05883	−1.76799	−0.58497
H	−5.02520	−2.19164	−0.83322
C	−3.53661	−1.91245	0.69788
H	−4.09526	−2.45026	1.45566
C	−2.29144	−1.36921	1.01909
H	−1.89433	−1.48918	2.01790
C	−0.18093	1.44101	0.20833
C	−0.67570	2.24398	1.24402
H	−1.00690	1.78480	2.16668
C	−0.73760	3.62712	1.10081
H	−1.11806	4.23309	1.91532
C	−0.31388	4.23145	−0.08329
H	−0.36236	5.30854	−0.19514
C	0.17495	3.43996	−1.11890
H	0.51298	3.89663	−2.04201
C	0.24264	2.05330	−0.97536
H	0.63800	1.46907	−1.79627

SCF energy: -1231.523077 hartree
zero-point correction: +0.297802 hartree
enthalpy correction: +0.316243 hartree
free energy correction: +0.250788 hartree
quasiharmonic free energy correction: +0.254987 hartree

3b (+*gauche*)

C	3.25012	−2.07857	0.44980
H	3.45812	−3.09855	0.12540
H	4.19136	−1.53557	0.53896
F	−0.05835	−0.15420	−1.91637
O	3.05760	0.18569	−1.02415
S	2.40341	−1.19407	−0.95887
C	2.37635	−2.00428	1.71674
H	2.98888	−1.79928	2.59676
H	1.88254	−2.96566	1.87941
C	1.31937	−0.91417	1.50308
H	1.75579	0.08171	1.61090
H	0.50945	−0.99485	2.23172
C	0.82611	−1.10299	0.06916
H	0.45375	−2.12416	−0.06662
C	−0.23909	−0.13141	−0.49344
C	−0.07382	1.32193	−0.04054
C	−0.67710	1.76253	1.14271
H	−1.27951	1.08153	1.73217
C	−0.52475	3.08044	1.57018
H	−0.99893	3.40377	2.48998
C	0.22085	3.98028	0.81017
H	0.33296	5.00832	1.13582
C	0.81747	3.54883	−0.37365
H	1.39920	4.24076	−0.97242
C	0.67841	2.22702	−0.79455
H	1.16433	1.89578	−1.70086
C	−1.67366	−0.62157	−0.26849
C	−2.69210	−0.01927	−1.02109
H	−2.44201	0.75809	−1.73280
C	−4.01682	−0.41375	−0.86954
H	−4.78892	0.05785	−1.46680
C	−4.35215	−1.40945	0.05026
H	−5.38513	−1.71443	0.17275
C	−3.35013	−2.00682	0.80822
H	−3.59675	−2.78032	1.52651
C	−2.01763	−1.61906	0.64602
H	−1.26405	−2.10590	1.25139

SCF energy: -1231.513727 hartree
zero-point correction: +0.297848 hartree
enthalpy correction: +0.316321 hartree
free energy correction: +0.251095 hartree
quasiharmonic free energy correction: +0.254670 hartree

3b (*anti*)

C	0.86298	3.60904	-0.38656
H	1.80957	4.14109	-0.29334
H	0.34106	3.95749	-1.27897
F	-0.13517	-0.41841	2.24734
O	0.42340	1.54432	-2.08711
S	1.24153	1.83100	-0.82352
C	-0.00713	3.66532	0.87867
H	-0.70012	4.50772	0.82515
H	0.62407	3.81641	1.75842
C	-0.74463	2.32733	1.00014
H	-1.55933	2.26032	0.27612
H	-1.17221	2.18971	1.99594
C	0.31584	1.26450	0.72049
H	1.12308	1.37981	1.45413
C	-0.07574	-0.23234	0.82081
C	1.05320	-1.14424	0.33238
C	0.96802	-1.85449	-0.86774
H	0.07427	-1.79077	-1.47381
C	2.03145	-2.64916	-1.29685
H	1.94848	-3.19270	-2.23111
C	3.18836	-2.75062	-0.52824
H	4.01175	-3.37279	-0.86018
C	3.27832	-2.05022	0.67506
H	4.17131	-2.12789	1.28491
C	2.22119	-1.25028	1.10058
H	2.29817	-0.72157	2.04256
C	-1.45603	-0.61415	0.29128
C	-2.25747	-1.48434	1.04057
H	-1.90779	-1.84955	1.99675
C	-3.50693	-1.88141	0.56771
H	-4.11542	-2.55205	1.16411
C	-3.97175	-1.42095	-0.66323
H	-4.94421	-1.72893	-1.03075
C	-3.17470	-0.56154	-1.41665
H	-3.52328	-0.19861	-2.37725
C	-1.92537	-0.15697	-0.94481
H	-1.31200	0.50456	-1.54724

SCF energy: -1231.516473 hartree
zero-point correction: +0.297953 hartree
enthalpy correction: +0.316357 hartree
free energy correction: +0.251260 hartree
quasiharmonic free energy correction: +0.255051 hartree

3b (*-gauche*)

C	3.29740	−2.02355	−0.46403
H	3.99949	−2.02467	−1.29760
H	3.84257	−2.17589	0.46922
F	0.05670	−0.23037	1.78736
O	3.00413	0.13702	1.15853
S	2.63771	−0.28758	−0.26389
C	2.12748	−2.99860	−0.63200
H	2.39558	−3.98461	−0.24573
H	1.88500	−3.11846	−1.69177
C	0.92723	−2.40453	0.10998
H	1.07112	−2.46174	1.19179
H	0.00140	−2.93035	−0.13262
C	0.85562	−0.94054	−0.32626
H	0.64471	−0.89674	−1.39925
C	−0.17652	−0.06172	0.40428
C	−1.59781	−0.58118	0.12466
C	−2.14172	−0.50975	−1.16311
H	−1.57555	−0.07214	−1.97664
C	−3.42450	−0.98820	−1.41841
H	−3.82946	−0.92425	−2.42199
C	−4.18673	−1.53980	−0.38842
H	−5.18681	−1.90818	−0.58629
C	−3.65288	−1.60897	0.89588
H	−4.23581	−2.03313	1.70573
C	−2.36499	−1.13605	1.15232
H	−1.95970	−1.19828	2.15285
C	−0.07554	1.43563	0.10526
C	−0.36859	2.35490	1.11866
H	−0.61315	1.99862	2.11060
C	−0.33393	3.72393	0.86530
H	−0.55638	4.42115	1.66516
C	−0.01285	4.19708	−0.40658
H	0.01537	5.26274	−0.60291
C	0.27506	3.28838	−1.42204
H	0.53010	3.64209	−2.41451
C	0.24435	1.91707	−1.16870
H	0.47835	1.23778	−1.97908

SCF energy: -1231.51739 hartree
zero-point correction: +0.298042 hartree
enthalpy correction: +0.316417 hartree
free energy correction: +0.251382 hartree
quasiharmonic free energy correction: +0.255118 hartree

4 (+*gauche*)

F	-0.12473	-0.25872	-1.81980
O	2.70833	0.38654	-1.28380
O	2.65758	-2.15640	-1.29543
S	2.45213	-0.88215	-0.59461
C	3.40331	-0.90696	0.97828
H	4.26950	-1.54491	0.80951
H	3.71787	0.12175	1.15781
C	2.42184	-1.41015	2.03316
H	2.76296	-1.15057	3.03753
H	2.34089	-2.49992	1.98428
C	1.06142	-0.75604	1.72412
H	1.11270	0.31586	1.92963
H	0.27264	-1.16809	2.35672
C	0.75603	-0.98576	0.23998
H	0.52699	-2.03614	0.05257
C	-0.35508	-0.13416	-0.42330
C	-1.75907	-0.71437	-0.20207
C	-2.81108	-0.13196	-0.92306
H	-2.60497	0.70227	-1.58255
C	-4.10770	-0.61740	-0.80810
H	-4.90544	-0.15797	-1.38070
C	-4.38330	-1.69058	0.04075
H	-5.39537	-2.06761	0.13412
C	-3.34856	-2.27497	0.76182
H	-3.54752	-3.11296	1.42027
C	-2.04348	-1.79301	0.63619
H	-1.26119	-2.28094	1.20270
C	-0.30979	1.35489	-0.07212
C	-0.95170	1.82807	1.07888
H	-1.50325	1.14329	1.71304
C	-0.91131	3.18052	1.40935
H	-1.41583	3.53031	2.30309
C	-0.24097	4.08119	0.58324
H	-0.21684	5.13605	0.83301
C	0.38950	3.61756	-0.56884
H	0.91022	4.31054	-1.22007
C	0.36183	2.26236	-0.89557
H	0.86635	1.90549	-1.78138

SCF energy: -1306.74643 hartree
zero-point correction: +0.303365 hartree
enthalpy correction: +0.322394 hartree
free energy correction: +0.256352 hartree
quasiharmonic free energy correction: +0.259658 hartree

4 (*anti*)

F	0.38665	0.54405	2.25837
O	-0.98279	-0.97830	-1.92473
O	-2.85000	-1.52688	-0.31276
S	-1.41915	-1.61313	-0.66569
C	-0.87304	-3.35978	-0.61379
H	-1.73523	-3.97176	-0.87343
H	-0.09803	-3.45334	-1.37531
C	-0.32780	-3.55986	0.79990
H	0.31211	-4.44264	0.83689
H	-1.14866	-3.71603	1.50444
C	0.46149	-2.28780	1.16207
H	1.38545	-2.24667	0.58152
H	0.73234	-2.27312	2.21869
C	-0.42689	-1.07990	0.84176
H	-1.22766	-1.01747	1.58005
C	0.24471	0.32407	0.84217
C	1.65514	0.38590	0.25909
C	2.68947	0.95149	1.01300
H	2.48924	1.32159	2.00887
C	3.98090	1.03981	0.49354
H	4.76811	1.47978	1.09538
C	4.25909	0.56726	-0.78688
H	5.26315	0.63535	-1.18993
C	3.23238	0.00790	-1.54565
H	3.43240	-0.35963	-2.54587
C	1.94057	-0.08288	-1.02930
H	1.15500	-0.50501	-1.64205
C	-0.69491	1.42836	0.35547
C	-0.39102	2.24589	-0.73588
H	0.53278	2.10654	-1.28052
C	-1.27337	3.24820	-1.13868
H	-1.02183	3.86980	-1.99041
C	-2.46647	3.45275	-0.45069
H	-3.15089	4.23305	-0.76362
C	-2.77345	2.64906	0.64739
H	-3.69608	2.80296	1.19515
C	-1.89643	1.64469	1.04584
H	-2.14538	1.04070	1.90972

SCF energy: -1306.74705 hartree
zero-point correction: +0.303543 hartree
enthalpy correction: +0.322515 hartree
free energy correction: +0.256780 hartree
quasiharmonic free energy correction: +0.260076 hartree

4 (*-gauche*)

F	0.08111	-0.30896	-1.77499
O	-2.69418	0.60440	-1.03509
O	-3.06034	0.06599	1.40997
S	-2.52562	-0.33144	0.08911
C	-3.16594	-1.98738	-0.33470
H	-4.18797	-2.03968	0.03715
H	-3.15228	-2.05428	-1.42340
C	-2.17714	-2.94543	0.32376
H	-2.28981	-3.95108	-0.08382
H	-2.36529	-3.00066	1.39910
C	-0.76539	-2.39537	0.04582
H	-0.51521	-2.54738	-1.00611
H	-0.01590	-2.91220	0.64552
C	-0.74507	-0.88998	0.36505
H	-0.61984	-0.73440	1.43596
C	0.32025	-0.07959	-0.39882
C	0.28230	1.43081	-0.17207
C	0.74418	2.27300	-1.19046
H	1.06433	1.84866	-2.13336
C	0.78090	3.65189	-1.00597
H	1.13310	4.29002	-1.80852
C	0.36659	4.21135	0.20330
H	0.39443	5.28541	0.34676
C	-0.08727	3.37915	1.22261
H	-0.41829	3.80077	2.16475
C	-0.12973	1.99649	1.03761
H	-0.50016	1.38060	1.84721
C	1.71413	-0.64304	-0.08043
C	2.25791	-0.49550	1.20082
H	1.71090	0.03492	1.97148
C	3.51511	-1.01447	1.49932
H	3.92188	-0.89134	2.49655
C	4.25094	-1.68045	0.51872
H	5.23200	-2.07931	0.74966
C	3.71709	-1.82368	-0.75949
H	4.28132	-2.33563	-1.53086
C	2.45357	-1.31179	-1.05897
H	2.04836	-1.42988	-2.05463

SCF energy: -1306.751434 hartree
zero-point correction: +0.303262 hartree
enthalpy correction: +0.322321 hartree
free energy correction: +0.255949 hartree
quasiharmonic free energy correction: +0.259832 hartree

Data for the torsion angle rotations around X-C-C-Y calculated at the G09 B3LYP/6-311+G(d,p) level of theory										
Dihedral angle [degrees]	Difluoroethane Total Energy (+174689) [kcal/mol]	Ethanesulfide Total Energy (+386957) [kcal/mol]	Ethansulfide_anti plus deltaG to gauche	Ethanesulfide_gauche Total Energy (+386958) [kcal/mol]	Ethanesulfoxide Total Energy (+434147) [kcal/mol]	Ethanesulfoxide_gauche plus deltaG to anti	Ethanesulfoxide_gauche Total Energy (+434147) [kcal/mol]	Ethanesulfone Total Energy (+481356) [kcal/mol]	Ethanesulfone_anti plus deltaG to gauche	Ethanesulfone_gauche Total Energy (+481358) [kcal/mol]
	Difluoroethane	Ethanesulfide_anti	Ethansulfide_anti	Ethanesulfide_gauche	Ethanesulfoxide_anti	Ethanesulfoxide_gauche	Ethanesulfoxide_gauche	Ethanesulfone_anti	Ethanesulfone_anti	Ethanesulfone_gauche
0	7.810358297	5.874516686	6.904516686	5.924605173	6.619879957	4.817684955	4.167684955	7.995832785	9.625832785	7.525349241
5	7.70509546	5.720783761	6.750783761	5.862991897	6.674165176	4.727136589	4.077136589	7.905315795	9.535315795	7.350143568
10	7.395657976	5.386889721	6.416889721	5.659909115	6.590073883	4.540325129	3.890325129	7.669637032	9.299637032	7.031620373
15	6.899966886	4.894353122	5.924353122	5.329337111	6.381209856	4.272287583	3.622287583	7.289049382	8.919049382	6.623012542
20	6.247304296	4.300295526	5.330295526	4.899520086	6.065633446	3.942895924	3.292895924	6.785487441	8.415487441	6.172193402
25	5.476035507	3.655777381	4.685777381	4.408106102	5.671933358	3.576619884	2.926619884	6.185250132	7.815250132	5.732507536
30	4.63096407	3.007650428	4.037650428	3.884134414	5.238396457	3.216303301	2.566303301	5.517376083	7.147376083	5.353926662
35	3.75905217	2.395976758	3.425976758	3.356842574	4.800416789	2.890923938	2.240923938	4.816033815	6.446033815	5.060134872
40	2.906140003	1.851075746	2.881075746	2.853812761	4.386703541	2.616566117	1.966566117	4.120754544	5.750754544	4.865052836
45	2.113779992	1.394346722	2.424346722	2.38293277	4.022562915	2.40708462	1.75708462	3.463540627	5.093540627	4.788464046
50	1.417021054	1.038861963	2.068861963	1.937042717	3.72994143	2.252767484	1.602767484	2.868933333	4.498933333	4.829919206
55	0.843154204	0.790366318	1.820366318	1.649234857	3.525629985	**2.166743462**	**1.516743462**	2.360038816	3.990038816	4.866642945
60	0.41044687	0.647575904	1.677575904	**1.379963**	3.416388738	2.167046549	1.517046549	1.95650691	3.58650691	4.866277107
65	0.128374447	**0.603994114**	**1.633994114**	1.291702534	**3.391342324**	2.24258426	1.59258426	1.669556228	3.299556228	4.845656517
70	**-0.002330762**	0.652145428	1.682145428	1.281374355	3.424024274	2.354701382	1.704701382	1.505429236	3.135429236	4.808718798
75	0.010162953	0.779611433	1.809611433	1.393482065	3.500139921	2.5375451	1.8875451	**1.458568709**	**3.088568709**	4.765028449
80	0.150770889	0.976264752	2.006264752	1.613467443	3.599920207	2.766928688	2.116928688	1.503613223	3.133613223	**4.733025464**
85	0.39934058	1.233249973	2.263249973	1.917181413	3.716673997	3.032776853	2.382776853	1.611293853	3.241293853	4.740059218
90	0.730273402	1.522164132	2.552164132	2.269917681	3.846403428	3.308111079	2.658111079	1.774831614	3.404831614	4.788087541

95	1.112874102	1.856767885	2.886767885	2.634650048	3.97778572	3.574349554	2.924349554	1.943794195	3.573794195	4.873868089
100	1.513471774	2.161737502	3.191737502	2.977905274	4.106041132	3.817044501	3.167044501	2.124360681	3.754360681	4.988072308
105	1.897567202	2.456832002	3.486832002	3.269228442	4.20024976	4.011571191	3.361571191	2.293553557	3.923553557	5.113209625
110	2.23249475	2.69317718	3.72317718	3.477632505	4.244920907	4.141632575	3.491632575	2.415878377	4.045878377	5.2096365
115	2.491327358	2.833014535	3.863014535	3.578360957	4.20830008	4.201594873	3.551594873	2.469360384	4.099360384	5.218025047
120	2.656028522	2.866654691	3.896654691	3.563566793	4.083293912	4.189111826	3.539111826	2.46644874	4.09644874	5.129234335
125	2.718835947	2.784202453	3.814202453	3.435801467	3.88284165	4.106816465	3.456816465	2.375144225	4.005144225	4.936245044
130	2.68215488	2.595128193	3.625128193	3.20481522	3.602503036	3.96163273	3.31163273	2.201775273	3.831775273	4.674712262
135	2.557604911	2.318051373	3.348051373	2.888315743	3.26060574	3.769697654	3.119697654	1.960740089	3.590740089	4.375026275
140	2.363648628	1.975226618	3.005226618	2.505687433	2.879128282	3.551146763	2.901146763	1.671748746	3.301748746	4.056347458
145	2.121451296	1.595892106	2.625892106	2.079563302	2.484303697	3.324593871	2.674593871	1.360566157	2.990566157	3.735681318
150	1.852107903	1.213652224	2.243652224	1.639297616	2.10006708	3.115311922	2.465311922	1.048917329	2.678917329	3.432199526
155	1.577751965	0.858073966	1.888073966	1.212759957	1.741718995	2.945188449	2.295188449	0.751073083	2.381073083	3.161175035
160	1.322210016	0.550833392	1.580833392	0.824645958	1.425929859	2.823572088	2.173572088	0.483562018	2.113562018	2.93077802
165	1.10649735	0.306688271	1.336688271	0.49615352	1.169501867	2.755878873	2.105878873	0.266269911	1.896269911	2.744497432
170	0.944107932	0.133650016	1.163650016	0.241904869	0.977936395	**2.74425677**	**2.09425677**	0.1141409	1.7441409	2.604076494
175	0.842806563	0.03185668	1.06185668	0.072505541	0.855453443	2.780356136	2.130356136	0.026239369	1.656239369	2.509683377
180	**0.808168666**	**-0.00001**	**1.0299886098**	**2.31742E-05**	**0.807332249**	2.857690407	2.207690407	**-0.002720194**	**1.627279806**	**2.465715042**
185	0.842792758	0.035845758	1.065845758	0.039568195	0.831839005	2.964683287	2.314683287	0.026235604	1.656235604	2.478002933
190	0.944099147	0.1401849	1.1701849	0.184739381	0.926325621	3.096630965	2.446630965	0.11412082	1.74412082	2.54089068
195	1.106486055	0.314112963	1.344112963	0.420660362	1.089942448	3.255284191	2.605284191	0.266275558	1.896275558	2.65402876
200	1.322194328	0.558484615	1.588484615	0.733862276	1.313679077	3.431614361	2.781614361	0.483560763	2.113560763	2.819727036
205	1.577731257	0.867689921	1.897689921	1.103580206	1.577184749	3.619222759	2.969222759	0.751035432	2.381035432	3.030594701
210	1.852086568	1.228325906	2.258325906	1.501514101	1.863514196	3.800699134	3.150699134	1.048896621	2.678896621	3.268403231
215	2.121432471	1.61813167	2.64813167	1.894392151	2.15804833	3.95683291	3.30683291	1.360574315	2.990574315	3.49999126
220	2.363634822	2.00523475	3.03523475	2.248364612	2.435519226	4.072322896	3.422322896	1.671744981	3.301744981	3.695873998
225	2.557598636	2.353476795	3.383476795	2.537893103	2.666126457	4.134625176	3.484625176	1.960775229	3.590775229	3.822353558
230	2.682156135	2.630349046	3.660349046	2.745087955	2.823008852	4.124190949	3.474190949	2.201787823	3.831787823	3.841301834
235	2.718841595	2.809087597	3.839087597	2.855234697	2.884325316	4.024711867	3.374711867	2.375245881	4.005245881	3.732118946

240	2.656034169	2.869158454	3.899158454	2.855674581	2.839743276	3.831667355	3.181667355	2.466433679	4.096433679	3.492981979
245	2.491328613	2.80544114	3.83544114	2.741331683	2.685987133	3.539188315	2.889188315	2.469379209	4.099379209	3.137621466
250	2.232487219	2.633679239	3.663679239	2.518955494	2.429369006	3.162833844	2.512833844	2.415786133	4.045786133	2.690483924
255	1.897554024	2.364359065	3.394359065	2.20545865	2.09047246	2.743367589	2.093367589	2.293486414	3.923486414	2.190872156
260	1.513457969	2.05822479	3.08822479	1.822792062	1.699678996	2.310599387	1.660599387	2.124734049	3.754734049	1.685558208
265	1.112864062	1.73803242	2.76803242	1.393466377	1.283761934	1.88689054	1.23689054	1.943777252	3.573777252	1.217498245
270	0.73026901	1.411371055	2.441371055	0.965658638	0.881538385	1.503297748	0.853297748	1.774886207	3.404886207	0.803943756
275	0.399344972	1.131700111	2.161700111	0.592486896	0.52700995	1.170865805	0.520865805	1.611271263	3.241271263	0.456877037
280	0.150784067	0.893627399	1.923627399	0.301267895	0.245743877	0.90712419	0.25712419	1.503603183	3.133603183	0.191635046
285	0.01018115	0.723556637	1.753556637	0.107073785	0.064148275	0.729042641	0.079042641	**1.458567454**	**3.088567454**	0.029779011
290	**-0.002315074**	0.624364955	1.654364955	**0.01874241**	**-0.000260555**	**0.650104456**	**0.000104456**	1.505406645	3.135406645	**-0.000258614**
295	0.12838386	**0.60867408**	**1.63867408**	0.055016852	0.064273777	0.678634803	0.028634803	1.669534892	3.299534892	0.125454757
300	0.410456283	0.685638748	1.715638748	0.236336977	0.264557867	0.815105569	0.165105569	1.956323677	3.586323677	0.410640254
305	0.843169891	0.861187668	1.891187668	0.564526955	0.606917638	1.050473087	0.400473087	2.359926492	3.989926492	0.835294131
310	1.417039252	1.140545484	2.170545484	1.017426917	1.064081526	1.373691308	0.723691308	2.868970356	4.498970356	1.380454304
315	2.11379317	1.523134262	2.553134262	1.562523084	1.62113928	1.777696357	1.127696357	3.463504232	5.093504232	2.048615125
320	2.906146278	2.002092183	3.032092183	2.175926171	2.261968924	2.246417088	1.596417088	4.120657281	5.750657281	2.835220246
325	3.759055935	2.562967704	3.592967704	2.835717897	2.952075006	2.750959826	2.100959826	4.81603005	6.44603005	3.706301323
330	4.630970346	3.183090162	4.213090162	3.509134109	3.663424796	3.254932159	2.604932159	5.517375456	7.147375456	4.606456798
335	5.476041782	3.830742717	4.860742717	4.156425846	4.366008905	3.722784417	3.072784417	6.185257034	7.815257034	5.472018302
340	6.247305551	4.463672016	5.493672016	4.739916182	5.02190819	4.12742325	3.47742325	6.785478656	8.415478656	6.244191332
345	6.899957474	5.033693489	6.063693489	5.229539255	5.598862387	4.452125531	3.802125531	7.289033067	8.919033067	6.866446695
350	7.395612168	5.488649799	6.518649799	5.602043327	6.071728446	4.680687081	4.030687081	7.669600636	9.299600636	7.296924487
355	7.705117423	5.773511501	6.803511501	5.837595333	6.417749734	4.803299927	4.153299927	7.905310775	9.535310775	7.516835192
360	7.810393437	5.853559751	6.883559751	5.924593878	6.619889369	4.8176586	4.1676586	7.984629858	9.614629858	7.525355516

References

[1] H.-Y. Wu, C.-W. Chang and R.-J. Chein, *J. Org. Chem.,* 2013, **78**, 5788.

[2] Oxidations were conducted based on an adapted literature procedure: F. Wang, H. Wang, P. L. Polavarapu and C. J. Rizzo, *J. Org. Chem.*, 2001, **66**, 35074.

[3] D. Asanuma, Y. Takaoka, S. Namiki, K. Takikawa, M. Kamiya, T. Nagano, Y. Urano and K. Hirose, *Angew. Chem. Int. Ed.,* 2014, **53**, 6085.

[4] Adapted from known literature procedure: S. Raghavan and C. N. Kumar, *Tetrahedron Lett.*, 2006, **46**, 1585.

[5] Z.-X. Wang, Y. Tu, M. Frohn, J.-R. Zhang and Y. Shi, *J. Am. Chem. Soc.*, 1997, **119**, 11224.
